# Supplementary material for: A genome-wide overexpression screen reveals Mycobacterium smegmatis growth inhibitors encoded by mycobacteriophage Hammy
Source: G3 (Bethesda). 2023 Nov 2;13(12):jkad240. doi: 10.1093/g3journal/jkad240 (PMC10700055; doi:10.1093/g3journal/jkad240)
Supplement: jkad240_Supplementary_Data [file jkad240_supplementary_data.pdf]

Supplemental Figure 1: Systematic Screening Results. Shown are the results of representative cytotoxicity assays for the 94 Hammy genes screened in this study. Each strain was spotted in triplicate alongside *M. smegmatis*/pExTra-Fruitloop52 (+) and pExTra-Fruitloop52I70S (-) control strains on 7H11 Kan supplemented with 0, 10, or 100 ng/ml aTc. In all experiments, 10^{-1} to 10^{-5} dilutions are shown; in some experiments the undiluted sample was also spotted. Plates were monitored over 3 or 4 days at 37 ºC, with results shown to best illustrate effects on colony color and size. Colony color was scored using the indicated key.

Gene *1*; Score *0*

Images taken after 3 days at 37 ºC

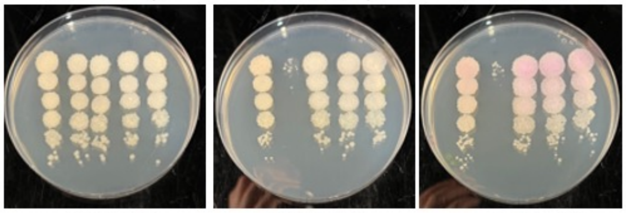

0 ng/ml aTc | 10 ng/ml aTc | 100 ng/ml aTc

Lane	Plasmid name	Gene name, replicate	Toxic/Non-toxic	Colony color on 100 ng/ml aTc plate*
- Non-toxic control	pExTra03	Fruitloop *52* mutant	Non-toxic	+
+ Toxic control	pExTra02	Fruitloop *52*	Toxic	-
1	pExTra-Hammy1	Hammy *1* replicate 1	Non-toxic	++
2	pExTra-Hammy1	Hammy *1* replicate 2	Non-toxic	++
3	pExTra-Hammy1	Hammy *1* replicate 3	Non-toxic	++

*Key: NG (no growth) - (no pink color) +(faint pink color) ++(obvious pink color) +++ (dark pink color)

Gene *2*; Score *0*

Images taken after 4 days at 37 ºC

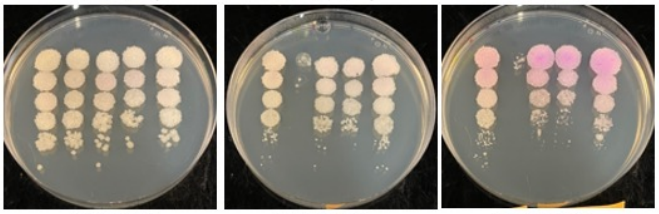

0 ng/ml aTc | 10 ng/ml aTc | 100 ng/ml aTc

Lane	Plasmid name	Gene name, replicate	Toxic/Non-toxic	Colony color on 100 ng/ml aTc plate*
- Non-toxic control	pExTra03	Fruitloop *52* mutant	Non-toxic	+
+ Toxic control	pExTra02	Fruitloop *52*	Toxic	-
1	pExTra-Hammy2	Hammy *2* replicate 1	Non-toxic	++
2	pExTra-Hammy2	Hammy *2* replicate 2	Non-toxic	++
3	pExTra-Hammy2	Hammy *2* replicate 3	Non-toxic	++

*Key: NG (no growth) - (no pink color) +(faint pink color) ++(obvious pink color) +++ (dark pink color)

Gene *3*; Score *0*

Images taken after 4 days at 37 ºC

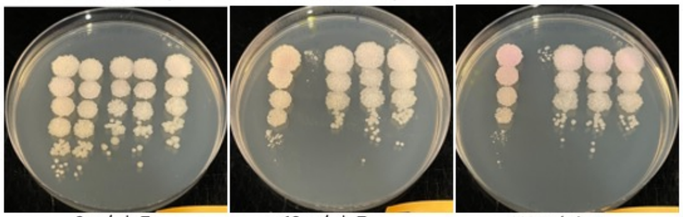

0 ng/ml aTc | 10 ng/ml aTc | 100 ng/ml aTc

Lane	Plasmid name	Gene name, replicate	Toxic/Non-toxic	Colony color on 100 ng/ml aTc plate*
- Non-toxic control	pExTra03	Fruitloop *52* mutant	Non-toxic	+
+ Toxic control	pExTra02	Fruitloop *52*	Toxic	-
1	pExTra-Hammy3	Hammy *3* replicate 1	Non-toxic	+
2	pExTra-Hammy3	Hammy *3* replicate 2	Non-toxic	+
3	pExTra-Hammy3	Hammy *3* replicate 3	Non-toxic	+

*Key: NG (no growth) - (no pink color) +(faint pink color) ++(obvious pink color) +++ (dark pink color)

Gene *5*; Score *0*

Images taken after 4 days at 37 ºC

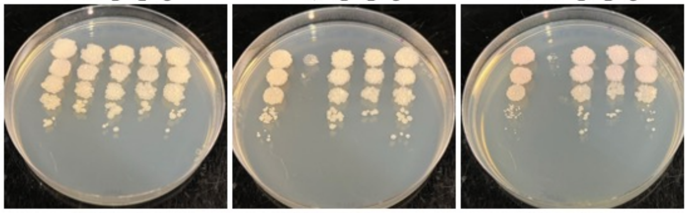

0 ng/ml aTc | 10 ng/ml aTc | 100 ng/ml aTc

Lane	Plasmid name	Gene name, replicate	Toxic/Non-toxic	Colony color on 100 ng/ml aTc plate*
- Non-toxic control	pExTra03	Fruitloop *52* mutant	Non-toxic	+
+ Toxic control	pExTra02	Fruitloop *52*	Toxic	-
1	pExTra-Hammy5	Hammy *5* replicate 1	Non-toxic	+
2	pExTra-Hammy5	Hammy *5* replicate 2	Non-toxic	+
3	pExTra-Hammy5	Hammy *5* replicate 3	Non-toxic	+

*Key: NG (no growth) - (no pink color) +(faint pink color) ++(obvious pink color) +++ (dark pink color)

Gene *6*; Score *0*

Images taken after 4 days at 37 ºC

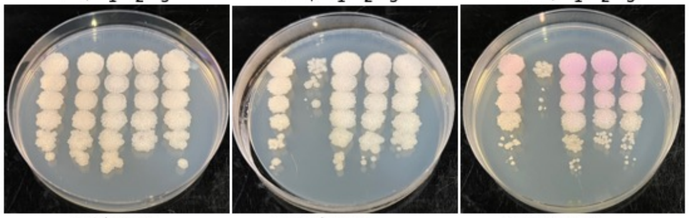

0 ng/ml aTc | 10 ng/ml aTc | 100 ng/ml aTc

Lane	Plasmid name	Gene name, replicate	Toxic/Non-toxic	Colony color on 100 ng/ml aTc plate*
- Non-toxic control	pExTra03	Fruitloop *52* mutant	Non-toxic	+
+ Toxic control	pExTra02	Fruitloop *52*	Toxic	-
1	pExTra-Hammy6	Hammy *6* replicate 1	Non-toxic	++
2	pExTra-Hammy6	Hammy *6* replicate 2	Non-toxic	++
3	pExTra-Hammy6	Hammy *6* replicate 3	Non-toxic	++

*Key: NG (no growth) - (no pink color) +(faint pink color) ++(obvious pink color) +++ (dark pink color)

Gene *7*; Score *0*

Images taken after 3 days at 37 ºC

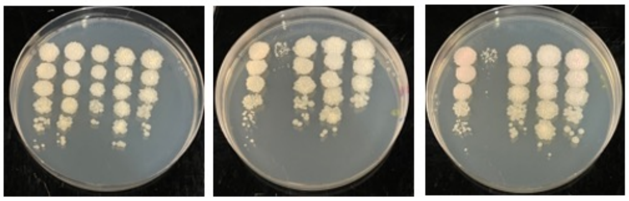

0 ng/ml aTc | 10 ng/ml aTc | 100 ng/ml aTc

Lane	Plasmid name	Gene name, replicate	Toxic/Non-toxic	Colony color on 100 ng/ml aTc plate*
- Non-toxic control	pExTra03	Fruitloop *52* mutant	Non-toxic	+
+ Toxic control	pExTra02	Fruitloop *52*	Toxic	-
1	pExTra-Hammy7	Hammy *7* replicate 1	Non-toxic	-
2	pExTra-Hammy7	Hammy *7* replicate 2	Non-toxic	-
3	pExTra-Hammy7	Hammy *7* replicate 3	Non-toxic	-

*Key: NG (no growth) - (no pink color) +(faint pink color) ++(obvious pink color) +++ (dark pink color)

Gene *8*; Score *0*

Images taken after 3 days at 37 ºC

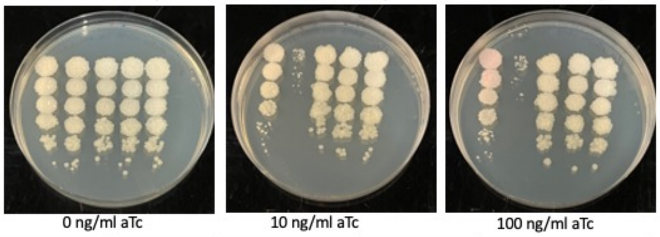

0 ng/ml aTc | 10 ng/ml aTc | 100 ng/ml aTc

Lane	Plasmid name	Gene name, replicate	Toxic/Non-toxic	Colony color on 100 ng/ml aTc plate*
- Non-toxic control	pExTra03	Fruitloop *52* mutant	Non-toxic	+
+ Toxic control	pExTra02	Fruitloop *52*	Toxic	-
1	pExTra-Hammy8	Hammy *8* replicate 1	Non-toxic	-
2	pExTra-Hammy8	Hammy *8* replicate 2	Non-toxic	-
3	pExTra-Hammy8	Hammy *8* replicate 3	Non-toxic	-

*Key: NG (no growth) - (no pink color) +(faint pink color) ++(obvious pink color) +++ (dark pink color)

Gene *9*; Score *3*

Images taken after 3 days at 37 ºC

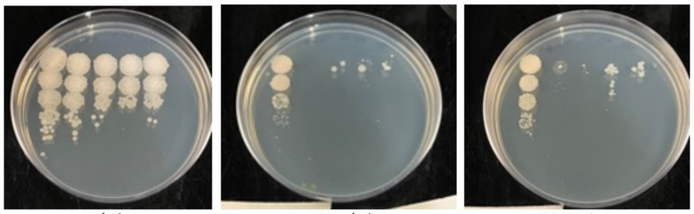

0 ng/ml aTc | 10 ng/ml aTc | 100 ng/ml aTc

Lane	Plasmid name	Gene name, replicate	Toxic/Non-toxic	Colony color on 100 ng/ml aTc plate*
- Non-toxic control	pExTra03	Fruitloop *52* mutant	Non-toxic	+
+ Toxic control	pExTra02	Fruitloop *52*	Toxic	-
1	pExTra-Hammy9	Hammy *9* replicate 1	Toxic	-/NG
2	pExTra-Hammy9	Hammy *9* replicate 2	Toxic	-/NG
3	pExTra-Hammy9	Hammy *9* replicate 3	Toxic	-/NG

*Key: NG (no growth) - (no pink color) +(faint pink color) ++(obvious pink color) +++ (dark pink color)

Gene *10*; Score *0*

Images taken after 4 days at 37 ºC

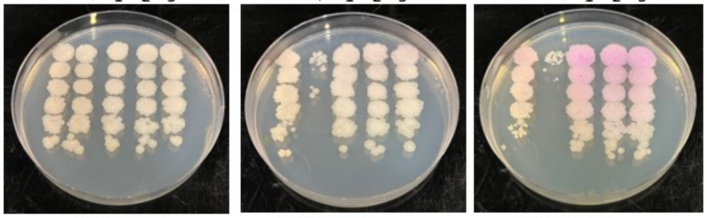

0 ng/ml aTc | 10 ng/ml aTc | 100 ng/ml aTc

Lane	Plasmid name	Gene name, replicate	Toxic/Non-toxic	Colony color on 100 ng/ml aTc plate*
- Non-toxic control	pExTra03	Fruitloop *52* mutant	Non-toxic	+
+ Toxic control	pExTra02	Fruitloop *52*	Toxic	-
1	pExTra-Hammy10	Hammy *10* replicate 1	Non-toxic	+++
2	pExTra-Hammy10	Hammy *10* replicate 2	Non-toxic	+++
3	pExTra-Hammy10	Hammy *10* replicate 3	Non-toxic	+++

*Key: NG (no growth) - (no pink color) +(faint pink color) ++(obvious pink color) +++ (dark pink color)

Gene *11*; Score *0*

Images taken after 3 days at 37 ºC

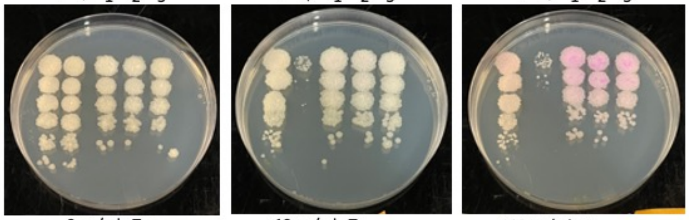

0 ng/ml aTc | 10 ng/ml aTc | 100 ng/ml aTc

Lane	Plasmid name	Gene name, replicate	Toxic/Non-toxic	Colony color on 100 ng/ml aTc plate*
- Non-toxic control	pExTra03	Fruitloop *52* mutant	Non-toxic	+
+ Toxic control	pExTra02	Fruitloop *52*	Toxic	-
1	pExTra-Hammy11	Hammy *11* replicate 1	Non-toxic	++
2	pExTra-Hammy11	Hammy *11* replicate 2	Non-toxic	++
3	pExTra-Hammy11	Hammy *11* replicate 3	Non-toxic	++

*Key: NG (no growth) - (no pink color) +(faint pink color) ++(obvious pink color) +++ (dark pink color)

Gene *12*; Score *1*

Images taken after 4 days at 37 ºC

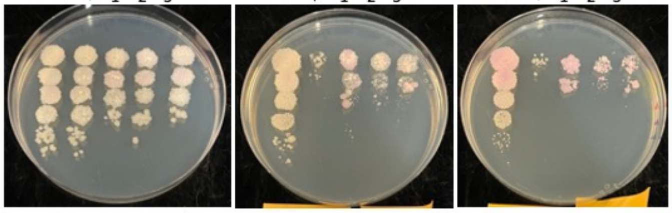

0 ng/ml aTc | 10 ng/ml aTc | 100 ng/ml aTc

Lane	Plasmid name	Gene name, replicate	Toxic/Non-toxic	Colony color on 100 ng/ml aTc plate*
- Non-toxic control	pExTra03	Fruitloop *52* mutant	Non-toxic	++
+ Toxic control	pExTra02	Fruitloop *52*	Toxic	-
1	pExTra-Hammy12	Hammy *12* replicate 1	Toxic	++
2	pExTra-Hammy12	Hammy *12* replicate 2	Toxic	++
3	pExTra-Hammy12	Hammy *12* replicate 3	Toxic	++

*Key: NG (no growth) - (no pink color) +(faint pink color) ++(obvious pink color) +++ (dark pink color)

Gene *13*; Score *0*

Images taken after 4 days at 37 ºC

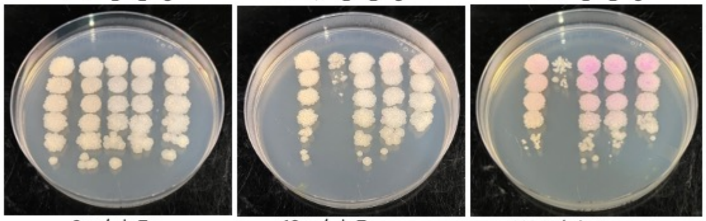

0 ng/ml aTc | 10 ng/ml aTc | 100 ng/ml aTc

Lane	Plasmid name	Gene name, replicate	Toxic/Non-toxic	Colony color on 100 ng/ml aTc plate*
- Non-toxic control	pExTra03	Fruitloop *52* mutant	Non-toxic	+
+ Toxic control	pExTra02	Fruitloop *52*	Toxic	-
1	pExTra-Hammy13	Hammy *13* replicate 1	Non-toxic	++
2	pExTra-Hammy13	Hammy *13* replicate 2	Non-toxic	++
3	pExTra-Hammy13	Hammy *13* replicate 3	Non-toxic	++

*Key: NG (no growth) - (no pink color) +(faint pink color) ++(obvious pink color) +++ (dark pink color)

Gene *14*; Score *0*

Images taken after 4 days at 37 ºC

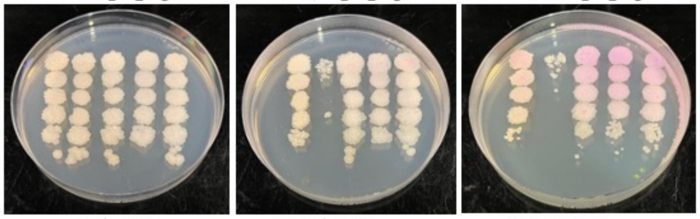

0 ng/ml aTc | 10 ng/ml aTc | 100 ng/ml aTc

Lane	Plasmid name	Gene name, replicate	Toxic/Non-toxic	Colony color on 100 ng/ml aTc plate*
- Non-toxic control	pExTra03	Fruitloop *52* mutant	Non-toxic	+
+ Toxic control	pExTra02	Fruitloop *52*	Toxic	-
1	pExTra-Hammy14	Hammy *14* replicate 1	Non-toxic	++
2	pExTra-Hammy14	Hammy *14* replicate 2	Non-toxic	++
3	pExTra-Hammy14	Hammy *14* replicate 3	Non-toxic	++

*Key: NG (no growth) - (no pink color) +(faint pink color) ++(obvious pink color) +++ (dark pink color)

Gene *15*; Score *0*

Images taken after 4 days at 37 ºC

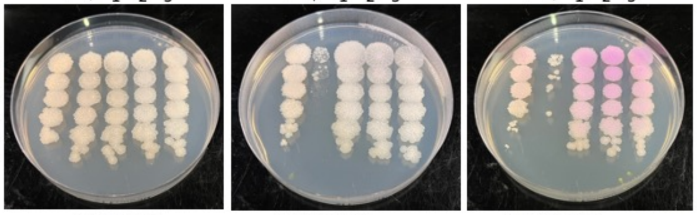

0 ng/ml aTc | 10 ng/ml aTc | 100 ng/ml aTc

Lane	Plasmid name	Gene name, replicate	Toxic/Non-toxic	Colony color on 100 ng/ml aTc plate*
- Non-toxic control	pExTra03	Fruitloop *52* mutant	Non-toxic	+
+ Toxic control	pExTra02	Fruitloop *52*	Toxic	-
1	pExTra-Hammy15	Hammy *15* replicate 1	Non-toxic	+++
2	pExTra-Hammy15	Hammy *15* replicate 2	Non-toxic	+++
3	pExTra-Hammy15	Hammy *15* replicate 3	Non-toxic	+++

*Key: NG (no growth) - (no pink color) +(faint pink color) ++(obvious pink color) +++ (dark pink color)

Gene *16*; Score *0*

Images taken after 4 days at 37 ºC

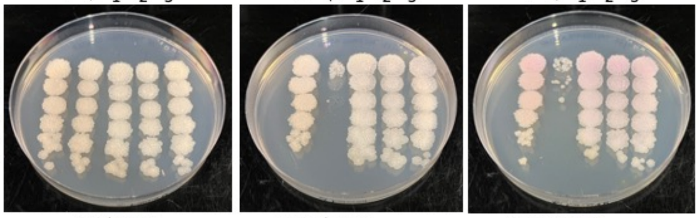

0 ng/ml aTc | 10 ng/ml aTc | 100 ng/ml aTc

Lane	Plasmid name	Gene name, replicate	Toxic/Non-toxic	Colony color on 100 ng/ml aTc plate*
- Non-toxic control	pExTra03	Fruitloop *52* mutant	Non-toxic	+
+ Toxic control	pExTra02	Fruitloop *52*	Toxic	-
1	pExTra-Hammy16	Hammy *16* replicate 1	Non-toxic	+
2	pExTra-Hammy16	Hammy *16* replicate 2	Non-toxic	+
3	pExTra-Hammy16	Hammy *16* replicate 3	Non-toxic	+

*Key: NG (no growth) - (no pink color) +(faint pink color) ++(obvious pink color) +++ (dark pink color)

Gene *17*; Score *0*

Images taken after 4 days at 37 ºC

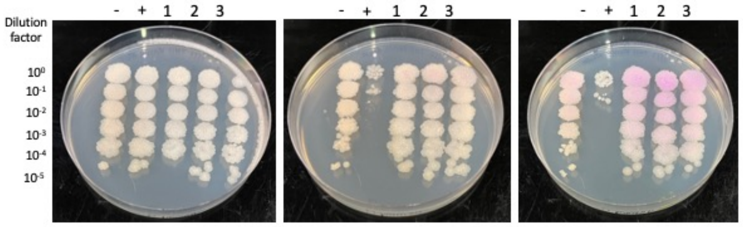

0 ng/ml aTc | 10 ng/ml aTc | 100 ng/ml aTc

Lane	Plasmid name	Gene name, replicate	Toxic/Non-toxic	Colony color on 100 ng/ml aTc plate*
- Non-toxic control	pExTra03	Fruitloop *52* mutant	Non-toxic	+
+ Toxic control	pExTra02	Fruitloop *52*	Toxic	-
1	pExTra-Hammy17	Hammy *17* replicate 1	Non-toxic	++
2	pExTra-Hammy17	Hammy *17* replicate 2	Non-toxic	++
3	pExTra-Hammy17	Hammy *17* replicate 3	Non-toxic	++

*Key: NG (no growth) - (no pink color) +(faint pink color) ++(obvious pink color) +++ (dark pink color)

Images taken after 4 days at 37 ºC

Gene *18*; Score *0*

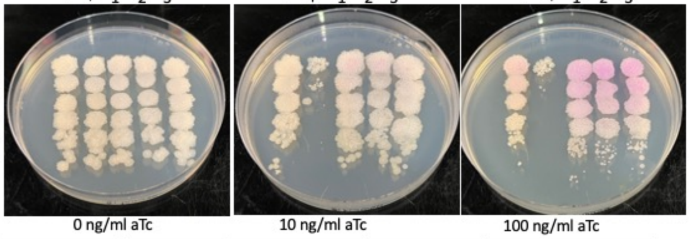

Lane	Plasmid name	Gene name, replicate	Toxic/Non-toxic	Colony color on 100 ng/ml aTc plate*
- Non-toxic control	pExTra03	Fruitloop *52* mutant	Non-toxic	+
+ Toxic control	pExTra02	Fruitloop *52*	Toxic	-
1	pExTra-Hammy18	Hammy *18* replicate 1	Non-toxic	++
2	pExTra-Hammy18	Hammy *18* replicate 2	Non-toxic	++
3	pExTra-Hammy18	Hammy *18* replicate 3	Non-toxic	++

*Key: NG (no growth) - (no pink color) +(faint pink color) ++(obvious pink color) +++ (dark pink color)

Images taken after 3 days at 37 ºC

Gene *20*; Score 3

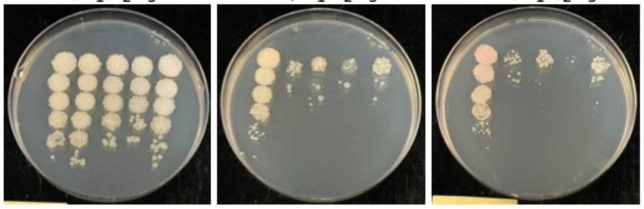

Lane	Plasmid name	Gene name, replicate	Toxic/Non-toxic	Colony color on 100 ng/ml aTc plate*
- Non-toxic control	pExTra03	Fruitloop *52* mutant	Non-toxic	+
+ Toxic control	pExTra02	Fruitloop *52*	Toxic	-
1	pExTra-Hammy20	Hammy *20* replicate 1	Toxic	-
2	pExTra-Hammy20	Hammy *20* replicate 2	Toxic	-/NG
3	pExTra-Hammy20	Hammy *20* replicate 3	Toxic	-

*Key: NG (no growth) - (no pink color) +(faint pink color) ++(obvious pink color) +++ (dark pink color)

Images taken after 4 days at 37 ºC

Gene *21*; Score *0*

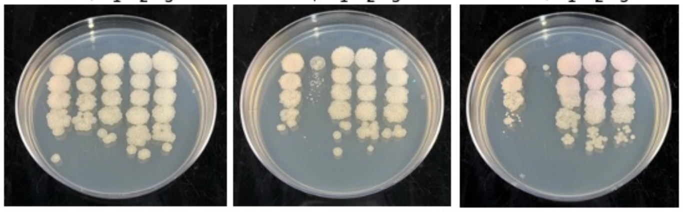

Lane	Plasmid name	Gene name, replicate	Toxic/Non-toxic	Colony color on 100 ng/ml aTc plate*
- Non-toxic control	pExTra03	Fruitloop *52* mutant	Non-toxic	+
+ Toxic control	pExTra02	Fruitloop *52*	Toxic	-
1	pExTra-Hammy21	Hammy *21* replicate 1	Non-toxic	+
2	pExTra-Hammy21	Hammy *21* replicate 2	Non-toxic	+
3	pExTra-Hammy21	Hammy *21* replicate 3	Non-toxic	+

*Key: NG (no growth) - (no pink color) +(faint pink color) ++(obvious pink color) +++ (dark pink color)

Images taken after 4 days at 37 ºC

Gene *22*; Score *0*

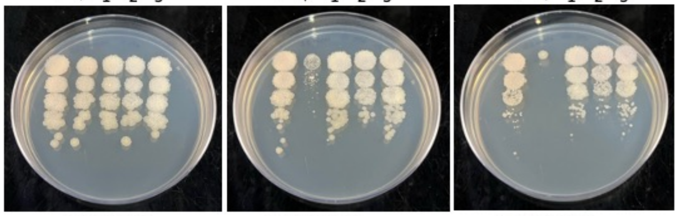

Lane	Plasmid name	Gene name, replicate	Toxic/Non-toxic	Colony color on 100 ng/ml aTc plate*
- Non-toxic control	pExTra03	Fruitloop *52* mutant	Non-toxic	-
+ Toxic control	pExTra02	Fruitloop *52*	Toxic	-
1	pExTra-Hammy22	Hammy *22* replicate 1	Non-toxic	-
2	pExTra-Hammy22	Hammy *22* replicate 2	Non-toxic	-
3	pExTra-Hammy22	Hammy *22* replicate 3	Non-toxic	-

*Key: NG (no growth) - (no pink color) +(faint pink color) ++(obvious pink color) +++ (dark pink color)

Images taken after 4 days at 37 ºC

Gene *23*; Score *0*

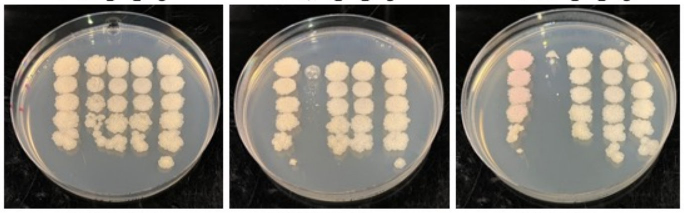

Lane	Plasmid name	Gene name, replicate	Toxic/Non-toxic	Colony color on 100 ng/ml aTc plate*
- Non-toxic control	pExTra03	Fruitloop *52* mutant	Non-toxic	+
+ Toxic control	pExTra02	Fruitloop *52*	Toxic	-
1	pExTra-Hammy23	Hammy *23* replicate 1	Non-toxic	-
2	pExTra-Hammy23	Hammy *23* replicate 2	Non-toxic	-
3	pExTra-Hammy23	Hammy *23* replicate 3	Non-toxic	-

*Key: NG (no growth) - (no pink color) +(faint pink color) ++(obvious pink color) +++ (dark pink color)

Images taken after 4 days at 37 ºC

Gene *24*; Score *0*

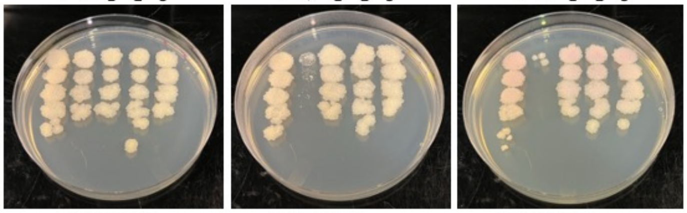

Lane	Plasmid name	Gene name, replicate	Toxic/Non-toxic	Colony color on 100 ng/ml aTc plate*
- Non-toxic control	pExTra03	Fruitloop *52* mutant	Non-toxic	+
+ Toxic control	pExTra02	Fruitloop *52*	Toxic	-
1	pExTra-Hammy24	Hammy *24* replicate 1	Non-toxic	+
2	pExTra-Hammy24	Hammy *24* replicate 2	Non-toxic	+
3	pExTra-Hammy24	Hammy *24* replicate 3	Non-toxic	+

*Key: NG (no growth) - (no pink color) +(faint pink color) ++(obvious pink color) +++ (dark pink color)

Images taken after 4 days at 37 ºC

Gene *25*; Score *0*

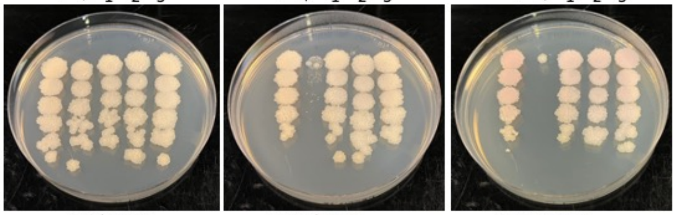

Lane	Plasmid name	Gene name, replicate	Toxic/Non-toxic	Colony color on 100 ng/ml aTc plate*
- Non-toxic control	pExTra03	Fruitloop *52* mutant	Non-toxic	+
+ Toxic control	pExTra02	Fruitloop *52*	Toxic	-
1	pExTra-Hammy25	Hammy *25* replicate 1	Non-toxic	+
2	pExTra-Hammy25	Hammy *25* replicate 2	Non-toxic	+
3	pExTra-Hammy25	Hammy *25* replicate 3	Non-toxic	+

*Key: NG (no growth) - (no pink color) +(faint pink color) ++(obvious pink color) +++ (dark pink color)

Images taken after 3 days at 37 ºC

Gene *26*; Score *0*

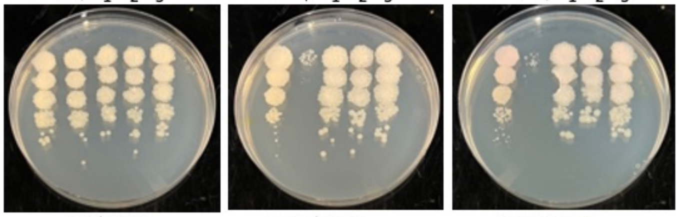

Lane	Plasmid name	Gene name, replicate	Toxic/Non-toxic	Colony color on 100 ng/ml aTc plate*
- Non-toxic control	pExTra03	Fruitloop *52* mutant	Non-toxic	+
+ Toxic control	pExTra02	Fruitloop *52*	Toxic	-
1	pExTra-Hammy26	Hammy *26* replicate 1	Non-toxic	+
2	pExTra-Hammy26	Hammy *26* replicate 2	Non-toxic	+
3	pExTra-Hammy26	Hammy *26* replicate 3	Non-toxic	+

*Key: NG (no growth) - (no pink color) +(faint pink color) ++(obvious pink color) +++ (dark pink color)

Gene *27*; Score *0*

Images taken after 3 days at 37 ºC

Dilution factor: 10^{0}, 10^{-1}, 10^{-2}, 10^{-3}, 10^{-4}, 10^{-5}

Lanes: - + 1 2 3

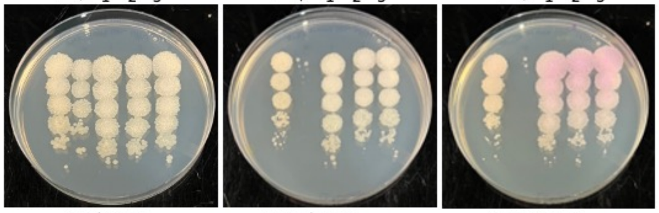

0 ng/ml aTc | 10 ng/ml aTc | 100 ng/ml aTc

Lane	Plasmid name	Gene name, replicate	Toxic/Non-toxic	Colony color on 100 ng/ml aTc plate*
- Non-toxic control	pExTra03	Fruitloop *52* mutant	Non-toxic	+
+ Toxic control	pExTra02	Fruitloop *52*	Toxic	-/NG
1	pExTra-Hammy27	Hammy *27* replicate 1	Non-toxic	++
2	pExTra-Hammy27	Hammy *27* replicate 2	Non-toxic	++
3	pExTra-Hammy27	Hammy *27* replicate 3	Non-toxic	++

*Key: NG (no growth) - (no pink color) +(faint pink color) ++(obvious pink color) +++ (dark pink color)

Gene *28*; Score *0*

Images taken after 4 days at 37 ºC

Dilution factor: 10^{0}, 10^{-1}, 10^{-2}, 10^{-3}, 10^{-4}, 10^{-5}

Lanes: - + 1 2 3

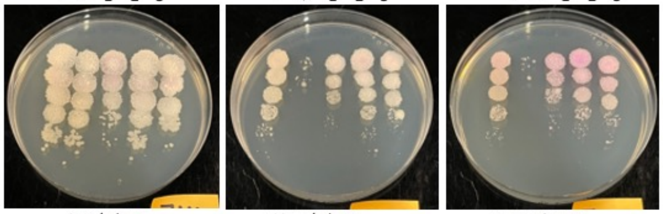

0 ng/ml aTc | 10 ng/ml aTc | 100 ng/ml aTc

Lane	Plasmid name	Gene name, replicate	Toxic/Non-toxic	Colony color on 100 ng/ml aTc plate*
- Non-toxic control	pExTra03	Fruitloop *52* mutant	Non-toxic	+
+ Toxic control	pExTra02	Fruitloop *52*	Toxic	-/NG
1	pExTra-Hammy28	Hammy *28* replicate 1	Non-toxic	++
2	pExTra-Hammy28	Hammy *28* replicate 2	Non-toxic	++
3	pExTra-Hammy28	Hammy *28* replicate 3	Non-toxic	++

*Key: NG (no growth) - (no pink color) +(faint pink color) ++(obvious pink color) +++ (dark pink color)

Gene *29*; Score *1*

Images taken after 3 days at 37 ºC

Dilution factor: 10^{0}, 10^{-1}, 10^{-2}, 10^{-3}, 10^{-4}, 10^{-5}

Lanes: - + 1 2 3

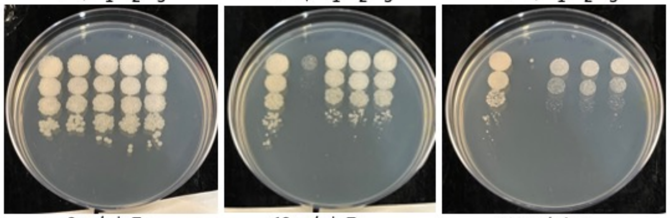

0 ng/ml aTc | 10 ng/ml aTc | 100 ng/ml aTc

Lane	Plasmid name	Gene name, replicate	Toxic/Non-toxic	Colony color on 100 ng/ml aTc plate*
- Non-toxic control	pExTra03	Fruitloop *52* mutant	Non-toxic	+
+ Toxic control	pExTra02	Fruitloop *52*	Toxic	-/NG
1	pExTra-Hammy29	Hammy *29* replicate 1	Toxic	-
2	pExTra-Hammy29	Hammy *29* replicate 2	Toxic	-
3	pExTra-Hammy29	Hammy *29* replicate 3	Toxic	-

*Key: NG (no growth) - (no pink color) +(faint pink color) ++(obvious pink color) +++ (dark pink color)

Gene *30*; Score *0*

Images taken after 4 days at 37 ºC

Dilution factor: 10^{0}, 10^{-1}, 10^{-2}, 10^{-3}, 10^{-4}, 10^{-5}

Lanes: - + 1 2 3

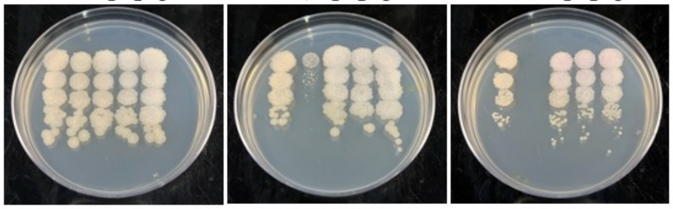

0 ng/ml aTc | 10 ng/ml aTc | 100 ng/ml aTc

Lane	Plasmid name	Gene name, replicate	Toxic/Non-toxic	Colony color on 100 ng/ml aTc plate*
- Non-toxic control	pExTra03	Fruitloop *52* mutant	Non-toxic	-
+ Toxic control	pExTra02	Fruitloop *52*	Toxic	NG
1	pExTra-Hammy30	Hammy *30* replicate 1	Non-toxic	+
2	pExTra-Hammy30	Hammy *30* replicate 2	Non-toxic	+
3	pExTra-Hammy30	Hammy *30* replicate 3	Non-toxic	+

*Key: NG (no growth) - (no pink color) +(faint pink color) ++(obvious pink color) +++ (dark pink color)

Gene *31*; Score *0*

Images taken after 4 days at 37 ºC

Dilution factor: 10^{0}, 10^{-1}, 10^{-2}, 10^{-3}, 10^{-4}, 10^{-5}

Lanes: - + 1 2 3

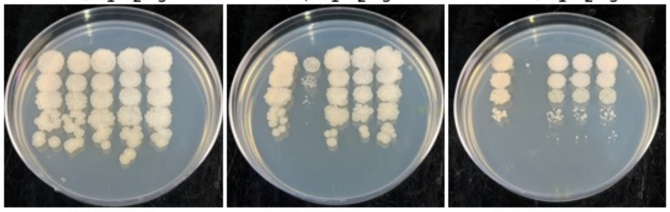

0 ng/ml aTc | 10 ng/ml aTc | 100 ng/ml aTc

Lane	Plasmid name	Gene name, replicate	Toxic/Non-toxic	Colony color on 100 ng/ml aTc plate*
- Non-toxic control	pExTra03	Fruitloop *52* mutant	Non-toxic	-
+ Toxic control	pExTra02	Fruitloop *52*	Toxic	-/NG
1	pExTra-Hammy31	Hammy *31* replicate 1	Non-toxic	-
2	pExTra-Hammy31	Hammy *31* replicate 2	Non-toxic	-
3	pExTra-Hammy31	Hammy *31* replicate 3	Non-toxic	-

*Key: NG (no growth) - (no pink color) +(faint pink color) ++(obvious pink color) +++ (dark pink color)

Gene *32*; Score *2*

Images taken after 4 days at 37 ºC

Dilution factor: 10^{0}, 10^{-1}, 10^{-2}, 10^{-3}, 10^{-4}, 10^{-5}

Lanes: - + 1 2 3

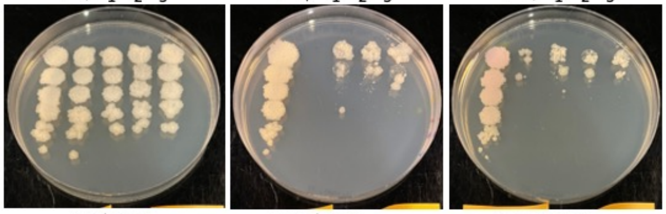

0 ng/ml aTc | 10 ng/ml aTc | 100 ng/ml aTc

Lane	Plasmid name	Gene name, replicate	Toxic/Non-toxic	Colony color on 100 ng/ml aTc plate*
- Non-toxic control	pExTra03	Fruitloop *52* mutant	Non-toxic	+
+ Toxic control	pExTra02	Fruitloop *52*	Toxic	-
1	pExTra-Hammy32	Hammy *32* replicate 1	Toxic	-
2	pExTra-Hammy32	Hammy *32* replicate 2	Toxic	-
3	pExTra-Hammy32	Hammy *32* replicate 3	Toxic	-

*Key: NG (no growth) - (no pink color) +(faint pink color) ++(obvious pink color) +++ (dark pink color)

Gene *33*; Score *0*

Images taken after 4 days at 37 ºC

Dilution factor: 10^{0}, 10^{-1}, 10^{-2}, 10^{-3}, 10^{-4}, 10^{-5}

Lanes: - + 1 2 3

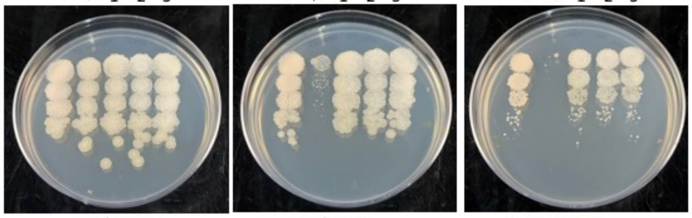

0 ng/ml aTc | 10 ng/ml aTc | 100 ng/ml aTc

Lane	Plasmid name	Gene name, replicate	Toxic/Non-toxic	Colony color on 100 ng/ml aTc plate*
- Non-toxic control	pExTra03	Fruitloop *52* mutant	Non-toxic	-
+ Toxic control	pExTra02	Fruitloop *52*	Toxic	-
1	pExTra-Hammy33	Hammy *33* replicate 1	Non-toxic	-
2	pExTra-Hammy33	Hammy *33* replicate 2	Non-toxic	-
3	pExTra-Hammy33	Hammy *33* replicate 3	Non-toxic	-

*Key: NG (no growth) - (no pink color) +(faint pink color) ++(obvious pink color) +++ (dark pink color)

Gene *34*; Score *3*

Images taken after 3 days at 37 ºC

Dilution factor: 10^{0}, 10^{-1}, 10^{-2}, 10^{-3}, 10^{-4}, 10^{-5}

Lanes: - + 1 2 3

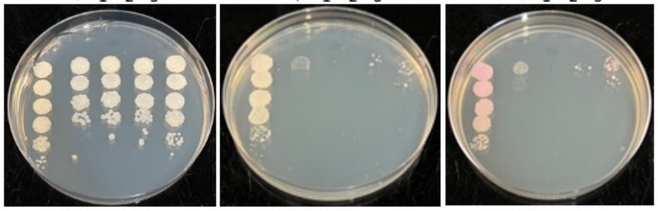

0 ng/ml aTc | 10 ng/ml aTc | 100 ng/ml aTc

Lane	Plasmid name	Gene name, replicate	Toxic/Non-toxic	Colony color on 100 ng/ml aTc plate*
- Non-toxic control	pExTra03	Fruitloop *52* mutant	Non-toxic	++
+ Toxic control	pExTra02	Fruitloop *52*	Toxic	-
1	pExTra-Hammy34	Hammy *34* replicate 1	Toxic	-/NG
2	pExTra-Hammy34	Hammy *34* replicate 2	Toxic	-/NG
3	pExTra-Hammy34	Hammy *34* replicate 3	Toxic	+

*Key: NG (no growth) - (no pink color) +(faint pink color) ++(obvious pink color) +++ (dark pink color)

Gene *35*; Score *0*

Images taken after 3 days at 37 ºC

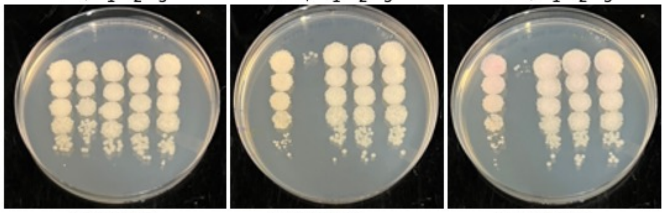

0 ng/ml aTc | 10 ng/ml aTc | 100 ng/ml aTc

Lane	Plasmid name	Gene name, replicate	Toxic/Non-toxic	Colony color on 100 ng/ml aTc plate*
- Non-toxic control	pExTra03	Fruitloop *52* mutant	Non-toxic	+
+ Toxic control	pExTra02	Fruitloop *52*	Toxic	-/NG
1	pExTra-Hammy35	Hammy *35* replicate 1	Non-toxic	-
2	pExTra-Hammy35	Hammy *35* replicate 2	Non-toxic	-
3	pExTra-Hammy35	Hammy *35* replicate 3	Non-toxic	-

*Key: NG (no growth) - (no pink color) +(faint pink color) ++(obvious pink color) +++ (dark pink color)

Gene *36*; Score *1*

Images taken after 3 days at 37 ºC

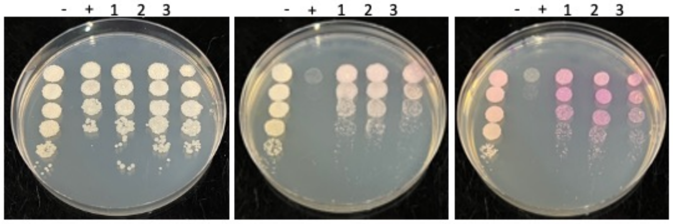

0 ng/ml aTc | 10 ng/ml aTc | 100 ng/ml aTc

Lane	Plasmid name	Gene name, replicate	Toxic/Non-toxic	Colony color on 100 ng/ml aTc plate*
- Non-toxic control	pExTra03	Fruitloop *52* mutant	Non-toxic	++
+ Toxic control	pExTra02	Fruitloop *52*	Toxic	-
1	pExTra-Hammy36	Hammy *36* replicate 1	Toxic	+++
2	pExTra-Hammy36	Hammy *36* replicate 2	Toxic	+++
3	pExTra-Hammy36	Hammy *36* replicate 3	Toxic	+++

*Key: NG (no growth) - (no pink color) +(faint pink color) ++(obvious pink color) +++ (dark pink color)

Gene *37*; Score *0*

Images taken after 4 days at 37 ºC

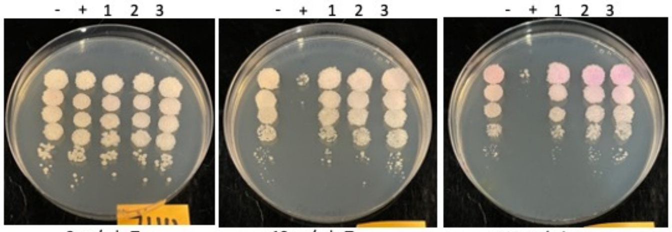

0 ng/ml aTc | 10 ng/ml aTc | 100 ng/ml aTc

Lane	Plasmid name	Gene name, replicate	Toxic/Non-toxic	Colony color on 100 ng/ml aTc plate*
- Non-toxic control	pExTra03	Fruitloop *52* mutant	Non-toxic	+
+ Toxic control	pExTra02	Fruitloop *52*	Toxic	-/NG
1	pExTra-Hammy37	Hammy *37* replicate 1	Non-toxic	++
2	pExTra-Hammy37	Hammy *37* replicate 2	Non-toxic	++
3	pExTra-Hammy37	Hammy *37* replicate 3	Non-toxic	++

*Key: NG (no growth) - (no pink color) +(faint pink color) ++(obvious pink color) +++ (dark pink color)

Gene *38*; Score *0*

Images taken after 4 days at 37 ºC

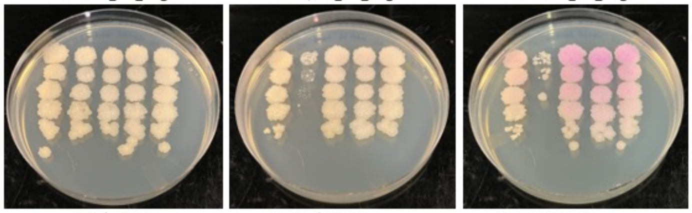

0 ng/ml aTc | 10 ng/ml aTc | 100 ng/ml aTc

Lane	Plasmid name	Gene name, replicate	Toxic/Non-toxic	Colony color on 100 ng/ml aTc plate*
- Non-toxic control	pExTra03	Fruitloop *52* mutant	Non-toxic	+
+ Toxic control	pExTra02	Fruitloop *52*	Toxic	-
1	pExTra-Hammy38	Hammy *38* replicate 1	Non-toxic	+++
2	pExTra-Hammy38	Hammy *38* replicate 2	Non-toxic	+++
3	pExTra-Hammy38	Hammy *38* replicate 3	Non-toxic	+++

*Key: NG (no growth) - (no pink color) +(faint pink color) ++(obvious pink color) +++ (dark pink color)

Gene *39*; Score *0*

Images taken after 4 days at 37 ºC

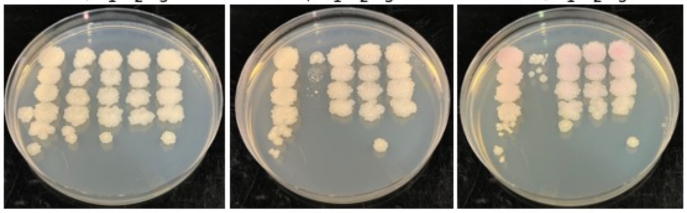

0 ng/ml aTc | 10 ng/ml aTc | 100 ng/ml aTc

Lane	Plasmid name	Gene name, replicate	Toxic/Non-toxic	Colony color on 100 ng/ml aTc plate*
- Non-toxic control	pExTra03	Fruitloop *52* mutant	Non-toxic	+
+ Toxic control	pExTra02	Fruitloop *52*	Toxic	-
1	pExTra-Hammy39	Hammy *39* replicate 1	Non-toxic	+
2	pExTra-Hammy39	Hammy *39* replicate 2	Non-toxic	+
3	pExTra-Hammy39	Hammy *39* replicate 3	Non-toxic	+

*Key: NG (no growth) - (no pink color) +(faint pink color) ++(obvious pink color) +++ (dark pink color)

Gene *40*; Score *0*

Images taken after 4 days at 37 ºC

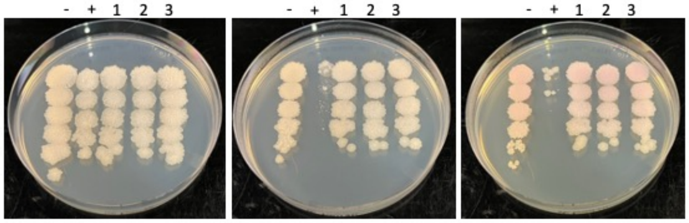

0 ng/ml aTc | 10 ng/ml aTc | 100 ng/ml aTc

Lane	Plasmid name	Gene name, replicate	Toxic/Non-toxic	Colony color on 100 ng/ml aTc plate*
- Non-toxic control	pExTra03	Fruitloop *52* mutant	Non-toxic	+
+ Toxic control	pExTra02	Fruitloop *52*	Toxic	-
1	pExTra-Hammy40	Hammy *40* replicate 1	Non-toxic	+
2	pExTra-Hammy40	Hammy *40* replicate 2	Non-toxic	+
3	pExTra-Hammy40	Hammy *40* replicate 3	Non-toxic	+

*Key: NG (no growth) - (no pink color) +(faint pink color) ++(obvious pink color) +++ (dark pink color)

Gene *41*; Score *0*

Images taken after 4 days at 37 ºC

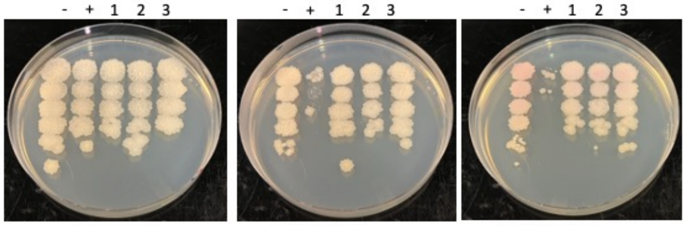

0 ng/ml aTc | 10 ng/ml aTc | 100 ng/ml aTc

Lane	Plasmid name	Gene name, replicate	Toxic/Non-toxic	Colony color on 100 ng/ml aTc plate*
- Non-toxic control	pExTra03	Fruitloop *52* mutant	Non-toxic	+
+ Toxic control	pExTra02	Fruitloop *52*	Toxic	-
1	pExTra-Hammy41	Hammy *41* replicate 1	Non-toxic	+
2	pExTra-Hammy41	Hammy *41* replicate 2	Non-toxic	+
3	pExTra-Hammy41	Hammy *41* replicate 3	Non-toxic	+

*Key: NG (no growth) - (no pink color) +(faint pink color) ++(obvious pink color) +++ (dark pink color)

Gene *42*; Score *0*

Images taken after 4 days at 37 ºC

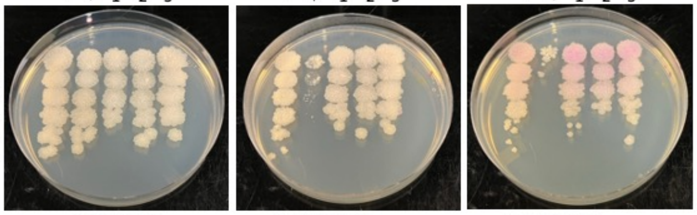

0 ng/ml aTc | 10 ng/ml aTc | 100 ng/ml aTc

Lane	Plasmid name	Gene name, replicate	Toxic/Non-toxic	Colony color on 100 ng/ml aTc plate*
- Non-toxic control	pExTra03	Fruitloop *52* mutant	Non-toxic	+
+ Toxic control	pExTra02	Fruitloop *52*	Toxic	-
1	pExTra-Hammy42	Hammy *42* replicate 1	Non-toxic	++
2	pExTra-Hammy42	Hammy *42* replicate 2	Non-toxic	++
3	pExTra-Hammy42	Hammy *42* replicate 3	Non-toxic	++

*Key: NG (no growth) - (no pink color) +(faint pink color) ++(obvious pink color) +++ (dark pink color)

Gene *43*; Score *0*

Images taken after 4 days at 37 ºC

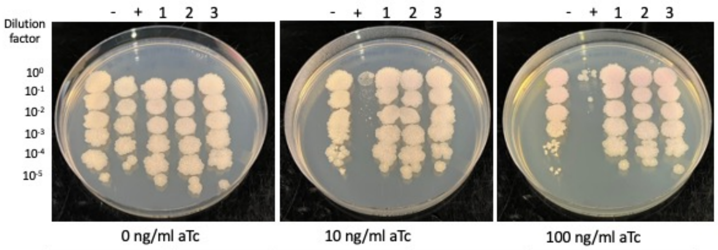

Lane	Plasmid name	Gene name, replicate	Toxic/Non-toxic	Colony color on 100 ng/ml aTc plate*
- Non-toxic control	pExTra03	Fruitloop *52* mutant	Non-toxic	+
+ Toxic control	pExTra02	Fruitloop *52*	Toxic	-
1	pExTra-Hammy43	Hammy *43* replicate 1	Non-toxic	+
2	pExTra-Hammy43	Hammy *43* replicate 2	Non-toxic	+
3	pExTra-Hammy43	Hammy *43* replicate 3	Non-toxic	+

*Key: NG (no growth) - (no pink color) +(faint pink color) ++(obvious pink color) +++ (dark pink color)

Gene *44*; Score *0*

Images taken after 4 days at 37 ºC

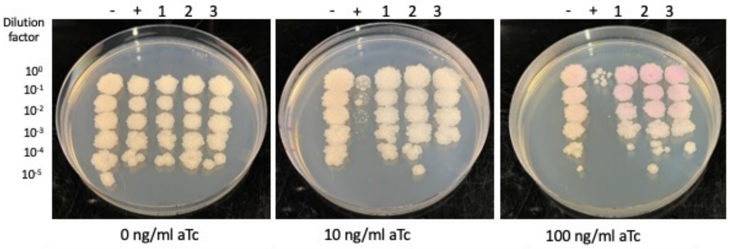

Lane	Plasmid name	Gene name, replicate	Toxic/Non-toxic	Colony color on 100 ng/ml aTc plate*
- Non-toxic control	pExTra03	Fruitloop *52* mutant	Non-toxic	+
+ Toxic control	pExTra02	Fruitloop *52*	Toxic	-
1	pExTra-Hammy44	Hammy *44* replicate 1	Non-toxic	++
2	pExTra-Hammy44	Hammy *44* replicate 2	Non-toxic	++
3	pExTra-Hammy44	Hammy *44* replicate 3	Non-toxic	++

*Key: NG (no growth) - (no pink color) +(faint pink color) ++(obvious pink color) +++ (dark pink color)

Gene *45*; Score *0*

Images taken after 4 days at 37 ºC

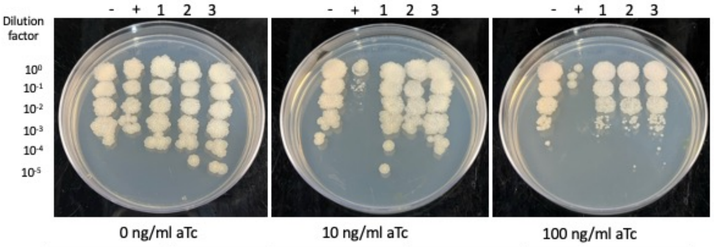

Lane	Plasmid name	Gene name, replicate	Toxic/Non-toxic	Colony color on 100 ng/ml aTc plate*
- Non-toxic control	pExTra03	Fruitloop *52* mutant	Non-toxic	+
+ Toxic control	pExTra02	Fruitloop *52*	Toxic	-
1	pExTra-Hammy45	Hammy *45* replicate 1	Non-toxic	-
2	pExTra-Hammy45	Hammy *45* replicate 2	Non-toxic	-
3	pExTra-Hammy45	Hammy *45* replicate 3	Non-toxic	-

*Key: NG (no growth) - (no pink color) +(faint pink color) ++(obvious pink color) +++ (dark pink color)

Gene *46*; Score *0*

Images taken after 4 days at 37 ºC

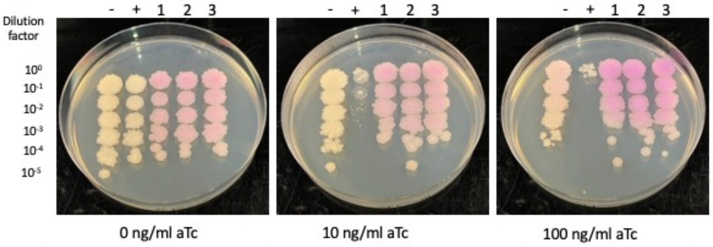

Lane	Plasmid name	Gene name, replicate	Toxic/Non-toxic	Colony color on 100 ng/ml aTc plate*
- Non-toxic control	pExTra03	Fruitloop *52* mutant	Non-toxic	+
+ Toxic control	pExTra02	Fruitloop *52*	Toxic	-
1	pExTra-Hammy46	Hammy *46* replicate 1	Non-toxic	+++
2	pExTra-Hammy46	Hammy *46* replicate 2	Non-toxic	+++
3	pExTra-Hammy46	Hammy *46* replicate 3	Non-toxic	+++

*Key: NG (no growth) - (no pink color) +(faint pink color) ++(obvious pink color) +++ (dark pink color)

Gene *47*; Score *0*

Images taken after 4 days at 37 ºC

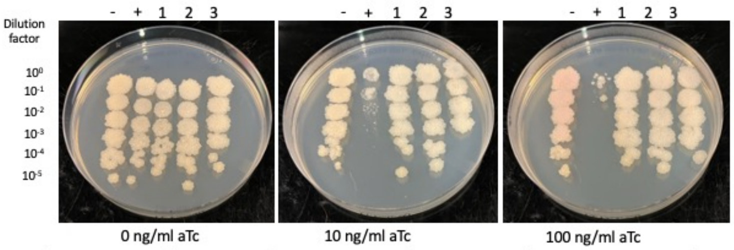

Lane	Plasmid name	Gene name, replicate	Toxic/Non-toxic	Colony color on 100 ng/ml aTc plate*
- Non-toxic control	pExTra03	Fruitloop *52* mutant	Non-toxic	+
+ Toxic control	pExTra02	Fruitloop *52*	Toxic	-
1	pExTra-Hammy47	Hammy *47* replicate 1	Non-toxic	-
2	pExTra-Hammy47	Hammy *47* replicate 2	Non-toxic	-
3	pExTra-Hammy47	Hammy *47* replicate 3	Non-toxic	-

*Key: NG (no growth) - (no pink color) +(faint pink color) ++(obvious pink color) +++ (dark pink color)

Gene *48*; Score *0*

Images taken after 3 days at 37 ºC

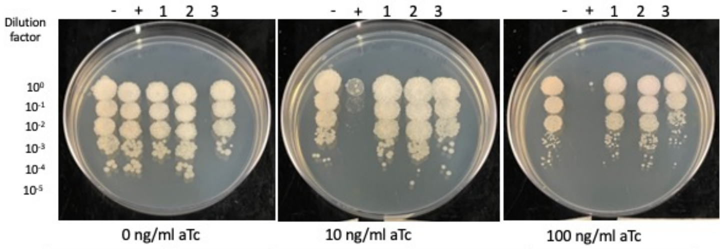

Lane	Plasmid name	Gene name, replicate	Toxic/Non-toxic	Colony color on 100 ng/ml aTc plate*
- Non-toxic control	pExTra03	Fruitloop *52* mutant	Non-toxic	-
+ Toxic control	pExTra02	Fruitloop *52*	Toxic	-/NG
1	pExTra-Hammy48	Hammy *48* replicate 1	Non-toxic	-
2	pExTra-Hammy48	Hammy *48* replicate 2	Non-toxic	-
3	pExTra-Hammy48	Hammy *48* replicate 3	Non-toxic	-

*Key: NG (no growth) - (no pink color) +(faint pink color) ++(obvious pink color) +++ (dark pink color)

Gene *49*; Score *0*

Images taken after 4 days at 37 ºC

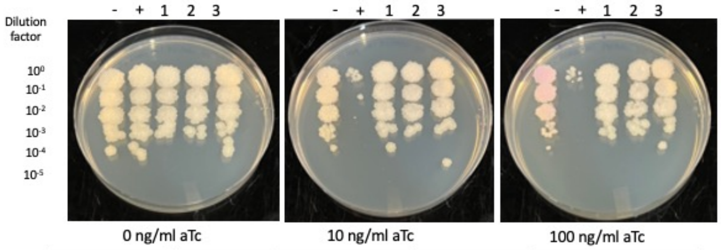

Lane	Plasmid name	Gene name, replicate	Toxic/Non-toxic	Colony color on 100 ng/ml aTc plate*
- Non-toxic control	pExTra03	Fruitloop *52* mutant	Non-toxic	++
+ Toxic control	pExTra02	Fruitloop *52*	Toxic	-
1	pExTra-Hammy49	Hammy *49* replicate 1	Non-toxic	-
2	pExTra-Hammy49	Hammy *49* replicate 2	Non-toxic	-
3	pExTra-Hammy49	Hammy 49 replicate 3	Non-toxic	-

*Key: NG (no growth) - (no pink color) +(faint pink color) ++(obvious pink color) +++ (dark pink color)

Gene *50*; Score *3*

Images taken after 4 days at 37 ºC

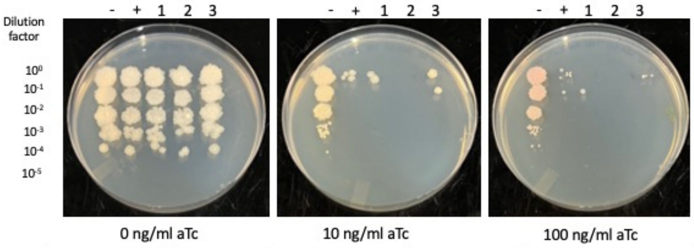

Lane	Plasmid name	Gene name, replicate	Toxic/Non-toxic	Colony color on 100 ng/ml aTc plate*
- Non-toxic control	pExTra03	Fruitloop *52* mutant	Non-toxic	+
+ Toxic control	pExTra02	Fruitloop *52*	Toxic	-/NG
1	pExTra-Hammy50	Hammy *50* replicate 1	Toxic	NG
2	pExTra-Hammy50	Hammy *50* replicate 2	Toxic	NG
3	pExTra-Hammy50	Hammy *50* replicate 3	Toxic	-/NG

*Key: NG (no growth) - (no pink color) +(faint pink color) ++(obvious pink color) +++ (dark pink color)

Images taken after 3 days at 37 ºC

Gene *51*; Score *3*

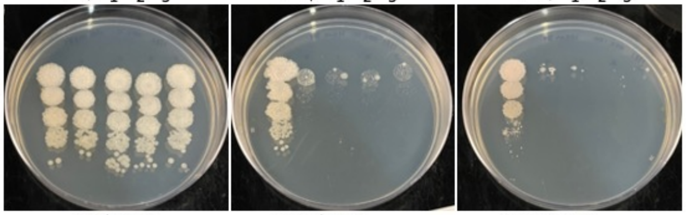

Lane	Plasmid name	Gene name, replicate	Toxic/Non-toxic	Colony color on 100 ng/ml aTc plate*
- Non-toxic control	pExTra03	Fruitloop *52* mutant	Non-toxic	+
+ Toxic control	pExTra02	Fruitloop *52*	Toxic	-
1	pExTra-Hammy51	Hammy *51* replicate 1	Toxic	-/NG
2	pExTra-Hammy51	Hammy *51* replicate 2	Toxic	NG
3	pExTra-Hammy51	Hammy *51* replicate 3	Toxic	NG

*Key: NG (no growth) - (no pink color) +(faint pink color) ++(obvious pink color) +++ (dark pink color)

Images taken after 4 days at 37 ºC

Gene *52*; Score *0*

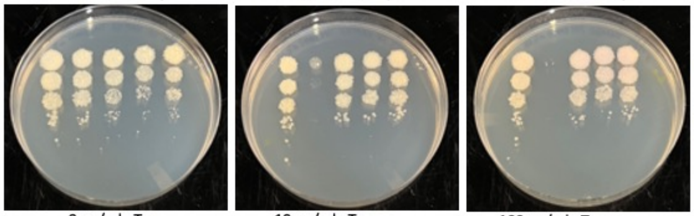

Lane	Plasmid name	Gene name, replicate	Toxic/Non-toxic	Colony color on 100 ng/ml aTc plate*
- Non-toxic control	pExTra03	Fruitloop *52* mutant	Non-toxic	-
+ Toxic control	pExTra02	Fruitloop *52*	Toxic	-
1	pExTra-Hammy52	Hammy *52* replicate 1	Non-toxic	+
2	pExTra-Hammy52	Hammy *52* replicate 2	Non-toxic	+
3	pExTra-Hammy52	Hammy *52* replicate 3	Non-toxic	+

*Key: NG (no growth) - (no pink color) +(faint pink color) ++(obvious pink color) +++ (dark pink color)

Images taken after 4 days at 37 ºC

Gene *53*; Score *1*

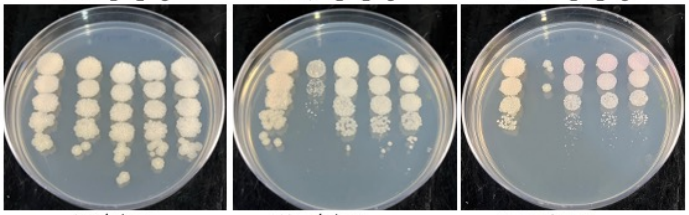

Lane	Plasmid name	Gene name, replicate	Toxic/Non-toxic	Colony color on 100 ng/ml aTc plate*
- Non-toxic control	pExTra03	Fruitloop *52* mutant	Non-toxic	+
+ Toxic control	pExTra02	Fruitloop *52*	Toxic	-
1	pExTra-Hammy53	Hammy *53* replicate 1	Toxic	++
2	pExTra-Hammy53	Hammy *53* replicate 2	Toxic	++
3	pExTra-Hammy53	Hammy *53* replicate 3	Toxic	++

*Key: NG (no growth) - (no pink color) +(faint pink color) ++(obvious pink color) +++ (dark pink color)

Images taken after 3 days at 37 ºC

Gene *54*; Score *3*

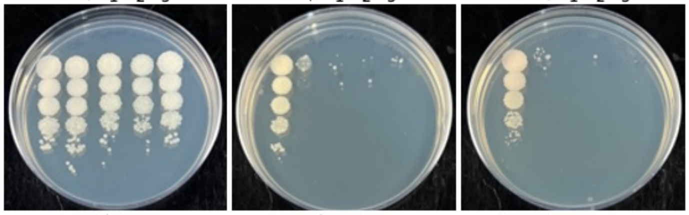

Lane	Plasmid name	Gene name, replicate	Toxic/Non-toxic	Colony color on 100 ng/ml aTc plate*
- Non-toxic control	pExTra03	Fruitloop *52* mutant	Non-toxic	+
+ Toxic control	pExTra02	Fruitloop *52*	Toxic	-
1	pExTra-Hammy54	Hammy *54* replicate 1	Toxic	NG
2	pExTra-Hammy54	Hammy *54* replicate 2	Toxic	NG
3	pExTra-Hammy54	Hammy *54* replicate 3	Toxic	NG

*Key: NG (no growth) - (no pink color) +(faint pink color) ++(obvious pink color) +++ (dark pink color)

Images taken after 4 days at 37 ºC

Gene *55*; Score *0*

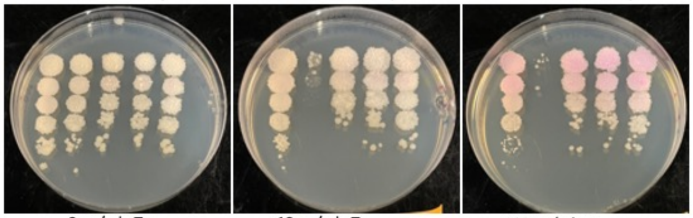

Lane	Plasmid name	Gene name, replicate	Toxic/Non-toxic	Colony color on 100 ng/ml aTc plate*
- Non-toxic control	pExTra03	Fruitloop *52* mutant	Non-toxic	++
+ Toxic control	pExTra02	Fruitloop *52*	Toxic	-
1	pExTra-Hammy55	Hammy *55* replicate 1	Non-toxic	++
2	pExTra-Hammy55	Hammy *55* replicate 2	Non-toxic	++
3	pExTra-Hammy55	Hammy *55* replicate 3	Non-toxic	++

*Key: NG (no growth) - (no pink color) +(faint pink color) ++(obvious pink color) +++ (dark pink color)

Images taken after 4 days at 37 ºC

Gene *56*; Score *3*

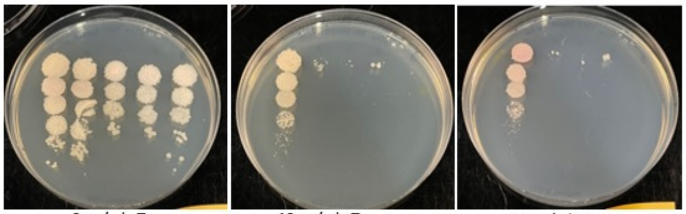

Lane	Plasmid name	Gene name, replicate	Toxic/Non-toxic	Colony color on 100 ng/ml aTc plate*
- Non-toxic control	pExTra03	Fruitloop *52* mutant	Non-toxic	+
+ Toxic control	pExTra02	Fruitloop *52*	Toxic	-/NG
1	pExTra-Hammy56	Hammy *56* replicate 1	Toxic	-/NG
2	pExTra-Hammy56	Hammy *56* replicate 2	Toxic	-/NG
3	pExTra-Hammy56	Hammy *56* replicate 3	Toxic	NG

*Key: NG (no growth) - (no pink color) +(faint pink color) ++(obvious pink color) +++ (dark pink color)

Images taken after 4 days at 37 ºC

Gene *57*; Score *0*

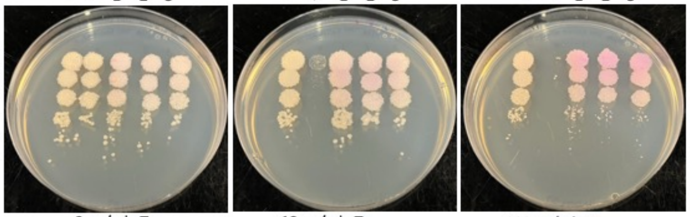

Lane	Plasmid name	Gene name, replicate	Toxic/Non-toxic	Colony color on 100 ng/ml aTc plate*
- Non-toxic control	pExTra03	Fruitloop *52* mutant	Non-toxic	+
+ Toxic control	pExTra02	Fruitloop *52*	Toxic	-
1	pExTra-Hammy57	Hammy *57* replicate 1	Non-toxic	++
2	pExTra-Hammy57	Hammy *57* replicate 2	Non-toxic	++
3	pExTra-Hammy57	Hammy *57* replicate 3	Non-toxic	++

*Key: NG (no growth) - (no pink color) +(faint pink color) ++(obvious pink color) +++ (dark pink color)

Images taken after 3 days at 37 ºC

Gene *58*; Score *3*

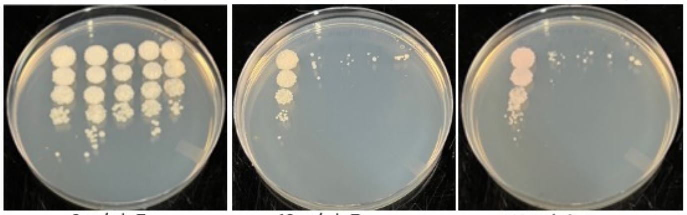

Lane	Plasmid name	Gene name, replicate	Toxic/Non-toxic	Colony color on 100 ng/ml aTc plate*
- Non-toxic control	pExTra03	Fruitloop *52* mutant	Non-toxic	+
+ Toxic control	pExTra02	Fruitloop *52*	Toxic	-/NG
1	pExTra-Hammy58	Hammy *58* replicate 1	Toxic	-/NG
2	pExTra-Hammy58	Hammy *58* replicate 2	Toxic	-/NG
3	pExTra-Hammy58	Hammy *58* replicate 3	Toxic	-/NG

*Key: NG (no growth) - (no pink color) +(faint pink color) ++(obvious pink color) +++ (dark pink color)

Images taken after 6 days at 37 ºC

Gene *59*; Score *0*

- + 1 2 3 (lanes, each plate)

Dilution factor: 10^{0}, 10^{-1}, 10^{-2}, 10^{-3}, 10^{-4}, 10^{-5}

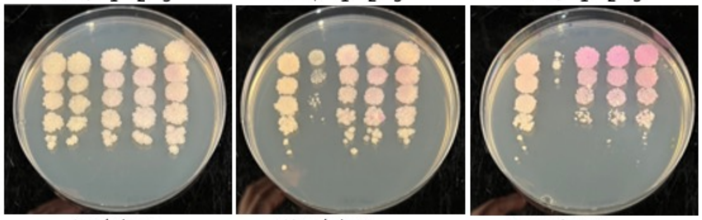

0 ng/ml aTc | 10 ng/ml aTc | 100 ng/ml aTc

Lane	Plasmid name	Gene name, replicate	Toxic/Non-toxic	Colony color on 100 ng/ml aTc plate*
- Non-toxic control	pExTra03	Fruitloop *52* mutant	Non-toxic	+
+ Toxic control	pExTra02	Fruitloop *52*	Toxic	-
1	pExTra-Hammy59	Hammy *59* replicate 1	Non-toxic	+++
2	pExTra-Hammy59	Hammy *59* replicate 2	Non-toxic	+++
3	pExTra-Hammy59	Hammy *59* replicate 3	Non-toxic	+++

*Key: NG (no growth) - (no pink color) +(faint pink color) ++(obvious pink color) +++ (dark pink color)

Images taken after 3 days at 37 ºC

Gene *60*; Score *1*

- + 1 2 3 (lanes, each plate)

Dilution factor: 10^{0}, 10^{-1}, 10^{-2}, 10^{-3}, 10^{-4}, 10^{-5}

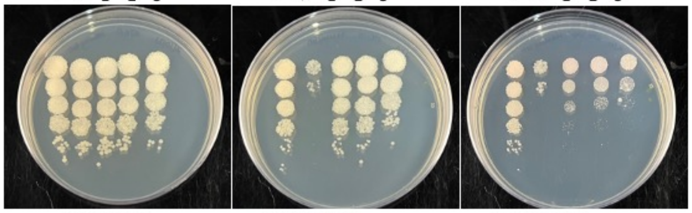

0 ng/ml aTc | 10 ng/ml aTc | 100 ng/ml aTc

Lane	Plasmid name	Gene name, replicate	Toxic/Non-toxic	Colony color on 100 ng/ml aTc plate*
- Non-toxic control	pExTra03	Fruitloop *52* mutant	Non-toxic	-
+ Toxic control	pExTra02	Fruitloop *52*	Toxic	-
1	pExTra-Hammy60	Hammy *60* replicate 1	Toxic	-
2	pExTra-Hammy60	Hammy *60* replicate 2	Toxic	-
3	pExTra-Hammy60	Hammy *60* replicate 3	Toxic	-

*Key: NG (no growth) - (no pink color) +(faint pink color) ++(obvious pink color) +++ (dark pink color)

Images taken after 3 days at 37 ºC

Gene *61*; Score *1*

- + 1 2 3 (lanes, each plate)

Dilution factor: 10^{0}, 10^{-1}, 10^{-2}, 10^{-3}, 10^{-4}, 10^{-5}

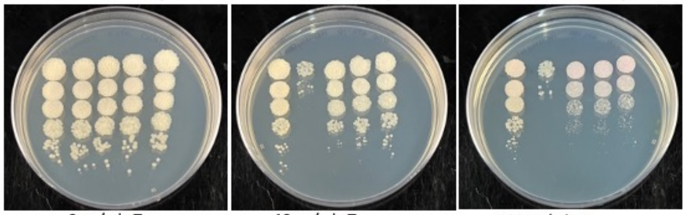

0 ng/ml aTc | 10 ng/ml aTc | 100 ng/ml aTc

Lane	Plasmid name	Gene name, replicate	Toxic/Non-toxic	Colony color on 100 ng/ml aTc plate*
- Non-toxic control	pExTra03	Fruitloop *52* mutant	Non-toxic	-
+ Toxic control	pExTra02	Fruitloop *52*	Toxic	-
1	pExTra-Hammy61	Hammy *61* replicate 1	Toxic	-
2	pExTra-Hammy61	Hammy *61* replicate 2	Toxic	-
3	pExTra-Hammy61	Hammy *61* replicate 3	Toxic	-

*Key: NG (no growth) - (no pink color) +(faint pink color) ++(obvious pink color) +++ (dark pink color)

Images taken after 3 days at 37 ºC

Gene *62*; Score *0*

- + 1 2 3 (lanes, each plate)

Dilution factor: 10^{0}, 10^{-1}, 10^{-2}, 10^{-3}, 10^{-4}, 10^{-5}

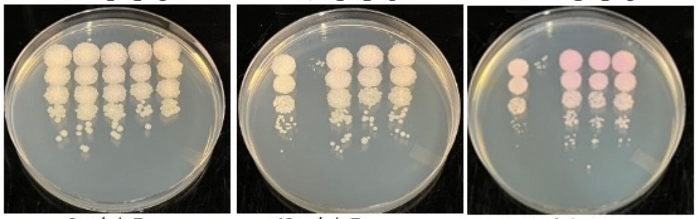

0 ng/ml aTc | 10 ng/ml aTc | 100 ng/ml aTc

Lane	Plasmid name	Gene name, replicate	Toxic/Non-toxic	Colony color on 100 ng/ml aTc plate*
- Non-toxic control	pExTra03	Fruitloop *52* mutant	Non-toxic	+
+ Toxic control	pExTra02	Fruitloop *52*	Toxic	-
1	pExTra-Hammy62	Hammy *62* replicate 1	Non-toxic	++
2	pExTra-Hammy62	Hammy *62* replicate 2	Non-toxic	++
3	pExTra-Hammy62	Hammy *62* replicate 3	Non-toxic	++

*Key: NG (no growth) - (no pink color) +(faint pink color) ++(obvious pink color) +++ (dark pink color)

Images taken after 3 days at 37 ºC

Gene *63*; Score *1*

- + 1 2 3 (lanes, each plate)

Dilution factor: 10^{0}, 10^{-1}, 10^{-2}, 10^{-3}, 10^{-4}, 10^{-5}

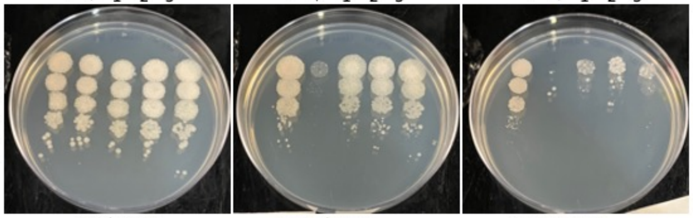

0 ng/ml aTc | 10 ng/ml aTc | 100 ng/ml aTc

Lane	Plasmid name	Gene name, replicate	Toxic/Non-toxic	Colony color on 100 ng/ml aTc plate*
- Non-toxic control	pExTra03	Fruitloop *52* mutant	Non-toxic	-
+ Toxic control	pExTra02	Fruitloop *52*	Toxic	-
1	pExTra-Hammy63	Hammy *63* replicate 1	Toxic	-
2	pExTra-Hammy63	Hammy *63* replicate 2	Toxic	-
3	pExTra-Hammy63	Hammy *63* replicate 3	Toxic	-

*Key: NG (no growth) - (no pink color) +(faint pink color) ++(obvious pink color) +++ (dark pink color)

Images taken after 4 days at 37 ºC

Gene *64*; Score *0*

- + 1 2 3 (lanes, each plate)

Dilution factor: 10^{0}, 10^{-1}, 10^{-2}, 10^{-3}, 10^{-4}, 10^{-5}

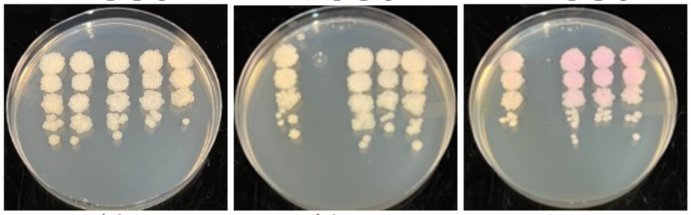

0 ng/ml aTc | 10 ng/ml aTc | 100 ng/ml aTc

Lane	Plasmid name	Gene name, replicate	Toxic/Non-toxic	Colony color on 100 ng/ml aTc plate*
- Non-toxic control	pExTra03	Fruitloop *52* mutant	Non-toxic	+
+ Toxic control	pExTra02	Fruitloop *52*	Toxic	-
1	pExTra-Hammy64	Hammy *64* replicate 1	Non-toxic	++
2	pExTra-Hammy64	Hammy *64* replicate 2	Non-toxic	++
3	pExTra-Hammy64	Hammy *64* replicate 3	Non-toxic	++

*Key: NG (no growth) - (no pink color) +(faint pink color) ++(obvious pink color) +++ (dark pink color)

Images taken after 3 days at 37 ºC

Gene *65*; Score *0*

- + 1 2 3 (lanes, each plate)

Dilution factor: 10^{0}, 10^{-1}, 10^{-2}, 10^{-3}, 10^{-4}, 10^{-5}

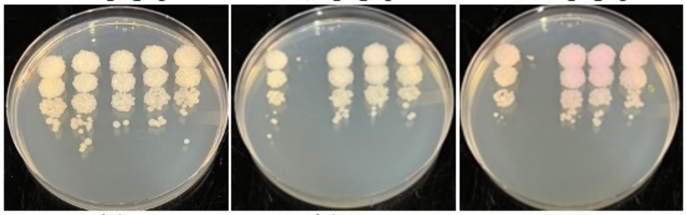

0 ng/ml aTc | 10 ng/ml aTc | 100 ng/ml aTc

Lane	Plasmid name	Gene name, replicate	Toxic/Non-toxic	Colony color on 100 ng/ml aTc plate*
- Non-toxic control	pExTra03	Fruitloop *52* mutant	Non-toxic	+
+ Toxic control	pExTra02	Fruitloop *52*	Toxic	-
1	pExTra-Hammy65	Hammy *65* replicate 1	Non-toxic	++
2	pExTra-Hammy65	Hammy *65* replicate 2	Non-toxic	++
3	pExTra-Hammy65	Hammy *65* replicate 3	Non-toxic	++

*Key: NG (no growth) - (no pink color) +(faint pink color) ++(obvious pink color) +++ (dark pink color)

Images taken after 3 days at 37 ºC

Gene *66*; Score *0*

- + 1 2 3 (lanes, each plate)

Dilution factor: 10^{0}, 10^{-1}, 10^{-2}, 10^{-3}, 10^{-4}, 10^{-5}

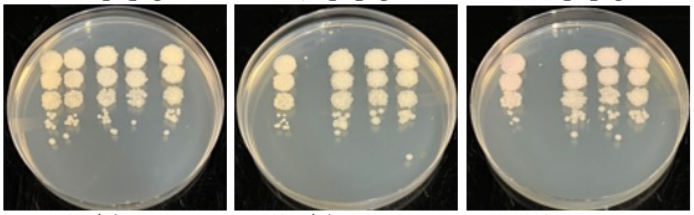

0 ng/ml aTc | 10 ng/ml aTc | 100 ng/ml aTc

Lane	Plasmid name	Gene name, replicate	Toxic/Non-toxic	Colony color on 100 ng/ml aTc plate*
- Non-toxic control	pExTra03	Fruitloop *52* mutant	Non-toxic	+
+ Toxic control	pExTra02	Fruitloop *52*	Toxic	-
1	pExTra-Hammy66	Hammy *66* replicate 1	Non-toxic	+
2	pExTra-Hammy66	Hammy *66* replicate 2	Non-toxic	+
3	pExTra-Hammy66	Hammy *66* replicate 3	Non-toxic	+

*Key: NG (no growth) - (no pink color) +(faint pink color) ++(obvious pink color) +++ (dark pink color)

Images taken after 4 days at 37 ºC

Gene *67*; Score *1*

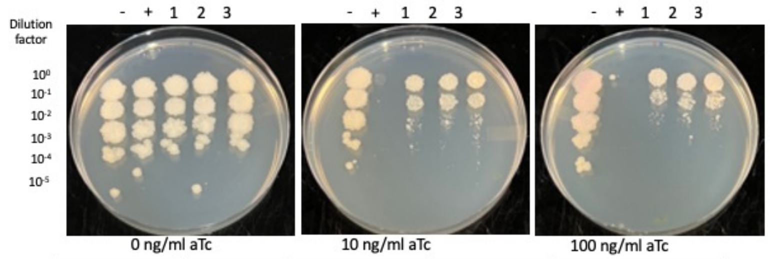

Lane	Plasmid name	Gene name, replicate	Toxic/Non-toxic	Colony color on 100 ng/ml aTc plate*
- Non-toxic control	pExTra03	Fruitloop *52* mutant	Non-toxic	+
+ Toxic control	pExTra02	Fruitloop *52*	Toxic	-
1	pExTra-Hammy67	Hammy *67* replicate 1	Toxic	-
2	pExTra-Hammy67	Hammy *67* replicate 2	Toxic	-
3	pExTra-Hammy67	Hammy *67* replicate 3	Toxic	-

*Key: NG (no growth) - (no pink color) +(faint pink color) ++(obvious pink color) +++ (dark pink color)

Images taken after 3 days at 37 ºC

Gene *68*; Score *3*

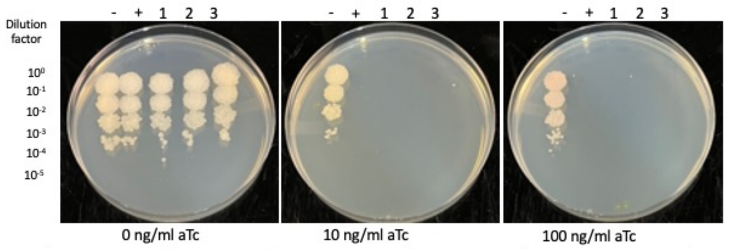

Lane	Plasmid name	Gene name, replicate	Toxic/Non-toxic	Colony color on 100 ng/ml aTc plate*
- Non-toxic control	pExTra03	Fruitloop *52* mutant	Non-toxic	+
+ Toxic control	pExTra02	Fruitloop *52*	Toxic	NG
1	pExTra-Hammy68	Hammy *68* replicate 1	Toxic	NG
2	pExTra-Hammy68	Hammy *68* replicate 2	Toxic	NG
3	pExTra-Hammy68	Hammy *68* replicate 3	Toxic	NG

*Key: NG (no growth) - (no pink color) +(faint pink color) ++(obvious pink color) +++ (dark pink color)

Images taken after 3 days at 37 ºC

Gene *69*; Score *3*

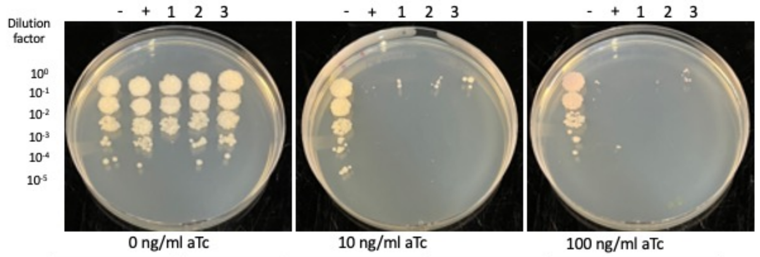

Lane	Plasmid name	Gene name, replicate	Toxic/Non-toxic	Colony color on 100 ng/ml aTc plate*
- Non-toxic control	pExTra03	Fruitloop *52* mutant	Non-toxic	+
+ Toxic control	pExTra02	Fruitloop *52*	Toxic	-/NG
1	pExTra-Hammy69	Hammy *69* replicate 1	Toxic	-/NG
2	pExTra-Hammy69	Hammy *69* replicate 2	Toxic	-/NG
3	pExTra-Hammy69	Hammy *69* replicate 3	Toxic	-/NG

*Key: NG (no growth) - (no pink color) +(faint pink color) ++(obvious pink color) +++ (dark pink color)

Images taken after 4 days at 37 ºC

Gene *70*; Score *0*

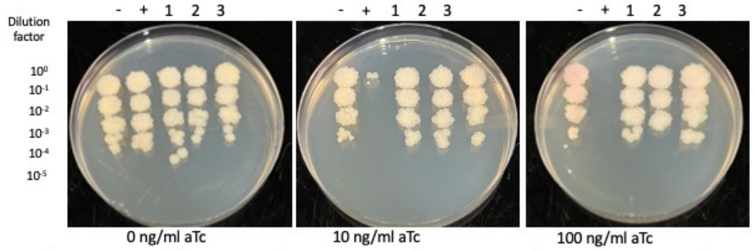

Lane	Plasmid name	Gene name, replicate	Toxic/Non-toxic	Colony color on 100 ng/ml aTc plate*
- Non-toxic control	pExTra03	Fruitloop *52* mutant	Non-toxic	+
+ Toxic control	pExTra02	Fruitloop *52*	Toxic	NG
1	pExTra-Hammy70	Hammy *70* replicate 1	Non-toxic	-
2	pExTra-Hammy70	Hammy *70* replicate 2	Non-toxic	-
3	pExTra-Hammy70	Hammy *70* replicate 3	Non-toxic	-

*Key: NG (no growth) - (no pink color) +(faint pink color) ++(obvious pink color) +++ (dark pink color)

Images taken after 5 days at 37 ºC

Gene *71*; Score *0*

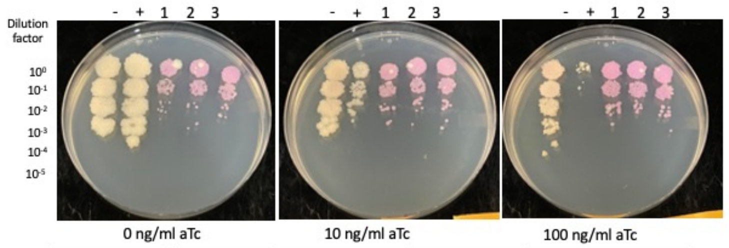

Lane	Plasmid name	Gene name, replicate	Toxic/Non-toxic	Colony color on 100 ng/ml aTc plate*
- Non-toxic control	pExTra03	Fruitloop *52* mutant	Non-toxic	+
+ Toxic control	pExTra02	Fruitloop *52*	Toxic	-
1	pExTra-Hammy71	Hammy *71* replicate 1	Non-toxic	+++
2	pExTra-Hammy71	Hammy *71* replicate 2	Non-toxic	+++
3	pExTra-Hammy71	Hammy *71* replicate 3	Non-toxic	+++

*Key: NG (no growth) - (no pink color) +(faint pink color) ++(obvious pink color) +++ (dark pink color)

Images taken after 4 days at 37 ºC

Gene *72*; Score *0*

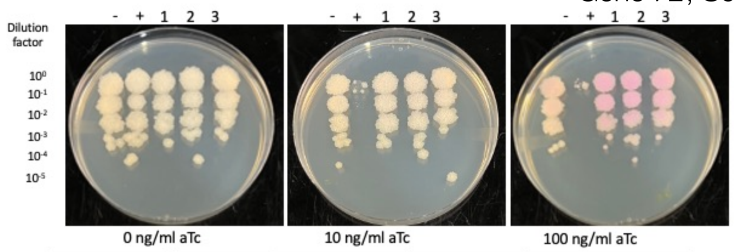

Lane	Plasmid name	Gene name, replicate	Toxic/Non-toxic	Colony color on 100 ng/ml aTc plate*
- Non-toxic control	pExTra03	Fruitloop *52* mutant	Non-toxic	+
+ Toxic control	pExTra02	Fruitloop *52*	Toxic	+
1	pExTra-Hammy72	Hammy *72* replicate 1	Non-toxic	++
2	pExTra-Hammy72	Hammy *72* replicate 2	Non-toxic	++
3	pExTra-Hammy72	Hammy *72* replicate 3	Non-toxic	++

*Key: NG (no growth) - (no pink color) +(faint pink color) ++(obvious pink color) +++ (dark pink color)

Images taken after 4 days at 37 ºC

Gene *73*; Score *0*

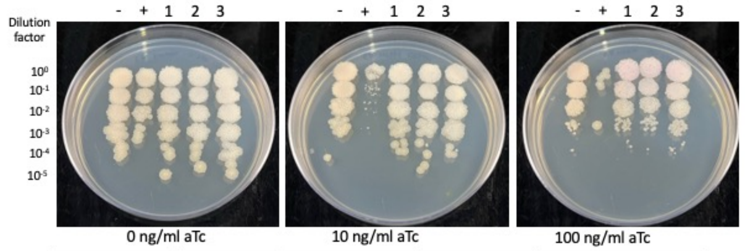

Lane	Plasmid name	Gene name, replicate	Toxic/Non-toxic	Colony color on 100 ng/ml aTc plate*
- Non-toxic control	pExTra03	Fruitloop *52* mutant	Non-toxic	+
+ Toxic control	pExTra02	Fruitloop *52*	Toxic	-
1	pExTra-Hammy73	Hammy *73* replicate 1	Non-toxic	+
2	pExTra-Hammy73	Hammy *73* replicate 2	Non-toxic	+
3	pExTra-Hammy73	Hammy *73* replicate 3	Non-toxic	+

*Key: NG (no growth) - (no pink color) +(faint pink color) ++(obvious pink color) +++ (dark pink color)

Images taken after 4 days at 37 ºC

Gene *74*; Score *0*

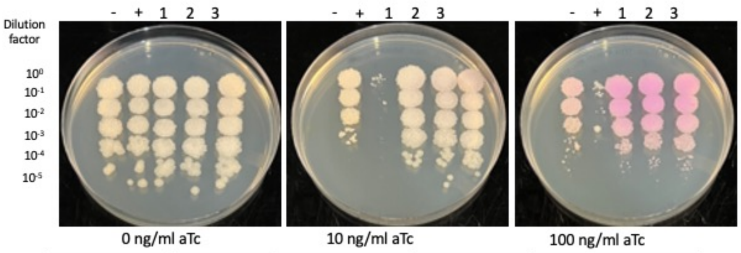

Lane	Plasmid name	Gene name, replicate	Toxic/Non-toxic	Colony color on 100 ng/ml aTc plate*
- Non-toxic control	pExTra03	Fruitloop *52* mutant	Non-toxic	++
+ Toxic control	pExTra02	Fruitloop *52*	Toxic	-
1	pExTra-Hammy74	Hammy *74* replicate 1	Non-toxic	+++
2	pExTra-Hammy74	Hammy *74* replicate 2	Non-toxic	+++
3	pExTra-Hammy74	Hammy *74* replicate 3	Non-toxic	+++

*Key: NG (no growth) - (no pink color) +(faint pink color) ++(obvious pink color) +++ (dark pink color)

Gene *75*; Score *0*

Images taken after 4 days at 37 ºC

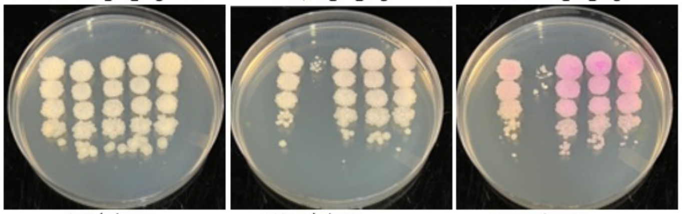

Lane	Plasmid name	Gene name, replicate	Toxic/Non-toxic	Colony color on 100 ng/ml aTc plate*
- Non-toxic control	pExTra03	Fruitloop *52* mutant	Non-toxic	++
+ Toxic control	pExTra02	Fruitloop *52*	Toxic	-
1	pExTra-Hammy75	Hammy *75* replicate 1	Non-toxic	+++
2	pExTra-Hammy75	Hammy *75* replicate 2	Non-toxic	+++
3	pExTra-Hammy75	Hammy *75* replicate 3	Non-toxic	+++

*Key: NG (no growth) - (no pink color) +(faint pink color) ++(obvious pink color) +++ (dark pink color)

Gene *76*; Score *0*

Images taken after 4 days at 37 ºC

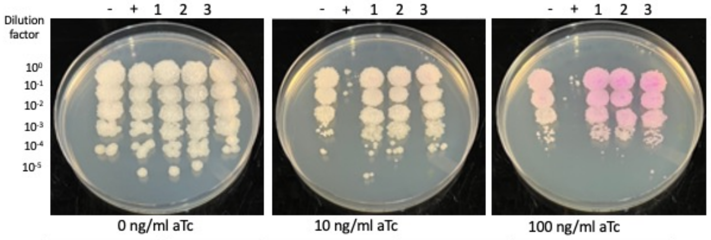

Lane	Plasmid name	Gene name, replicate	Toxic/Non-toxic	Colony color on 100 ng/ml aTc plate*
- Non-toxic control	pExTra03	Fruitloop *52* mutant	Non-toxic	+
+ Toxic control	pExTra02	Fruitloop *52*	Toxic	-
1	pExTra-Hammy76	Hammy *76* replicate 1	Non-toxic	+++
2	pExTra-Hammy76	Hammy *76* replicate 2	Non-toxic	+++
3	pExTra-Hammy76	Hammy *76* replicate 3	Non-toxic	+++

*Key: NG (no growth) - (no pink color) +(faint pink color) ++(obvious pink color) +++ (dark pink color)

Gene *77*; Score *3*

Images taken after 4 days at 37 ºC

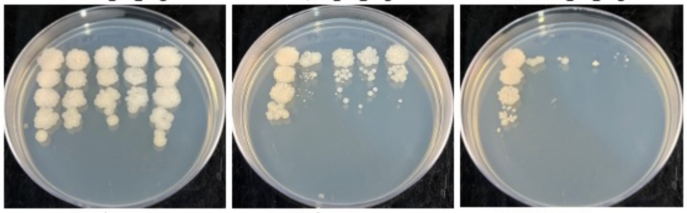

Lane	Plasmid name	Gene name, replicate	Toxic/Non-toxic	Colony color on 100 ng/ml aTc plate*
- Non-toxic control	pExTra03	Fruitloop *52* mutant	Non-toxic	+
+ Toxic control	pExTra02	Fruitloop *52*	Toxic	-/NG
1	pExTra-Hammy77	Hammy *77* replicate 1	Toxic	-/NG
2	pExTra-Hammy77	Hammy *77* replicate 2	Toxic	-/NG
3	pExTra-Hammy77	Hammy *77* replicate 3	Toxic	-/NG

*Key: NG (no growth) - (no pink color) +(faint pink color) ++(obvious pink color) +++ (dark pink color)

Gene *78*; Score *3*

Images taken after 4 days at 37 ºC

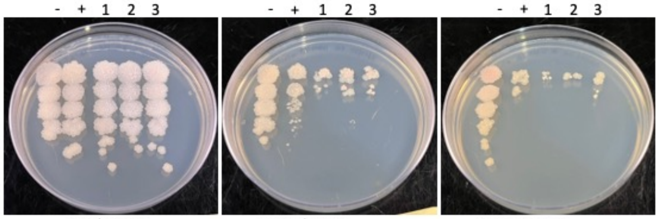

Lane	Plasmid name	Gene name, replicate	Toxic/Non-toxic	Colony color on 100 ng/ml aTc plate*
- Non-toxic control	pExTra03	Fruitloop *52* mutant	Non-toxic	+
+ Toxic control	pExTra02	Fruitloop *52*	Toxic	-/NG
1	pExTra-Hammy78	Hammy *78* replicate 1	Toxic	-/NG
2	pExTra-Hammy78	Hammy *78* replicate 2	Toxic	-/NG
3	pExTra-Hammy78	Hammy *78* replicate 3	Toxic	-/NG

*Key: NG (no growth) - (no pink color) +(faint pink color) ++(obvious pink color) +++ (dark pink color)

Gene *79*; Score *0*

Images taken after 4 days at 37 ºC

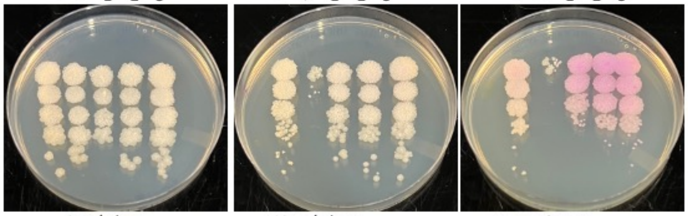

Lane	Plasmid name	Gene name, replicate	Toxic/Non-toxic	Colony color on 100 ng/ml aTc plate*
- Non-toxic control	pExTra03	Fruitloop *52* mutant	Non-toxic	+
+ Toxic control	pExTra02	Fruitloop *52*	Toxic	-
1	pExTra-Hammy79	Hammy *79* replicate 1	Non-toxic	+++
2	pExTra-Hammy79	Hammy *79* replicate 2	Non-toxic	+++
3	pExTra-Hammy79	Hammy *79* replicate 3	Non-toxic	+++

*Key: NG (no growth) - (no pink color) +(faint pink color) ++(obvious pink color) +++ (dark pink color)

Gene *80*; Score *0*

Images taken after 4 days at 37 ºC

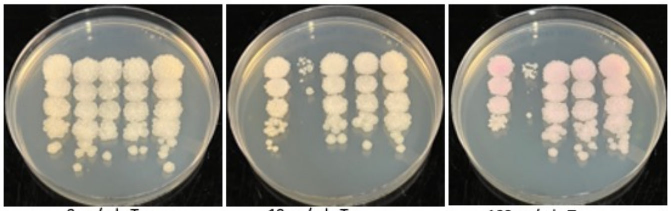

Lane	Plasmid name	Gene name, replicate	Toxic/Non-toxic	Colony color on 100 ng/ml aTc plate*
- Non-toxic control	pExTra03	Fruitloop *52* mutant	Non-toxic	+
+ Toxic control	pExTra02	Fruitloop *52*	Toxic	-
1	pExTra-Hammy80	Hammy *80* replicate 1	Non-toxic	+
2	pExTra-Hammy80	Hammy *80* replicate 2	Non-toxic	+
3	pExTra-Hammy80	Hammy *80* replicate 3	Non-toxic	+

*Key: NG (no growth) - (no pink color) +(faint pink color) ++(obvious pink color) +++ (dark pink color)

Gene *81*; Score *0*

Images taken after 4 days at 37 ºC

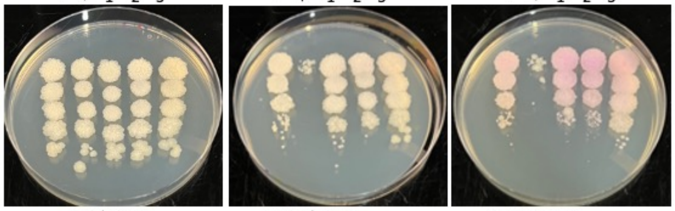

Lane	Plasmid name	Gene name, replicate	Toxic/Non-toxic	Colony color on 100 ng/ml aTc plate*
- Non-toxic control	pExTra03	Fruitloop *52* mutant	Non-toxic	+
+ Toxic control	pExTra02	Fruitloop *52*	Toxic	-
1	pExTra-Hammy81	Hammy *81* replicate 1	Non-toxic	++
2	pExTra-Hammy81	Hammy *81* replicate 2	Non-toxic	++
3	pExTra-Hammy81	Hammy *81* replicate 3	Non-toxic	+

*Key: NG (no growth) - (no pink color) +(faint pink color) ++(obvious pink color) +++ (dark pink color)

Gene *82*; Score *0*

Images taken after 3 days at 37 ºC

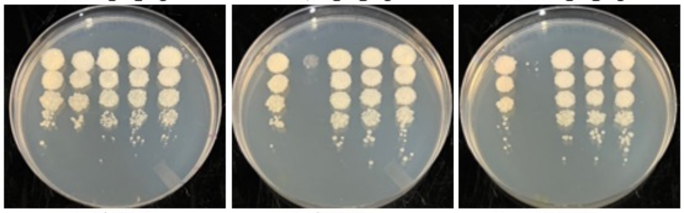

Lane	Plasmid name	Gene name, replicate	Toxic/Non-toxic	Colony color on 100 ng/ml aTc plate*
- Non-toxic control	pExTra03	Fruitloop *52* mutant	Non-toxic	-
+ Toxic control	pExTra02	Fruitloop *52*	Toxic	-
1	pExTra-Hammy82	Hammy *82* replicate 1	Non-toxic	-
2	pExTra-Hammy82	Hammy *82* replicate 2	Non-toxic	-
3	pExTra-Hammy82	Hammy *82* replicate 3	Non-toxic	-

*Key: NG (no growth) - (no pink color) +(faint pink color) ++(obvious pink color) +++ (dark pink color)

Gene *83*; Score *0*

Images taken after 3 days at 37 ºC

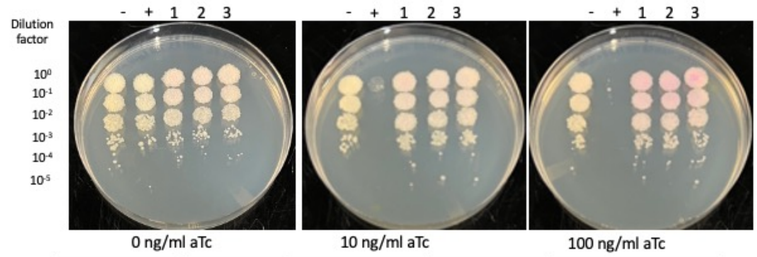

Lane	Plasmid name	Gene name, replicate	Toxic/Non-toxic	Colony color on 100 ng/ml aTc plate*
- Non-toxic control	pExTra03	Fruitloop *52* mutant	Non-toxic	-
+ Toxic control	pExTra02	Fruitloop *52*	Toxic	-
1	pExTra-Hammy83	Hammy *83* replicate 1	Non-toxic	++
2	pExTra-Hammy83	Hammy *83* replicate 2	Non-toxic	++
3	pExTra-Hammy83	Hammy *83* replicate 3	Non-toxic	++

*Key: NG (no growth) - (no pink color) +(faint pink color) ++(obvious pink color) +++ (dark pink color)

Gene *84*; Score *1*

Images taken after 5 days at 37 ºC

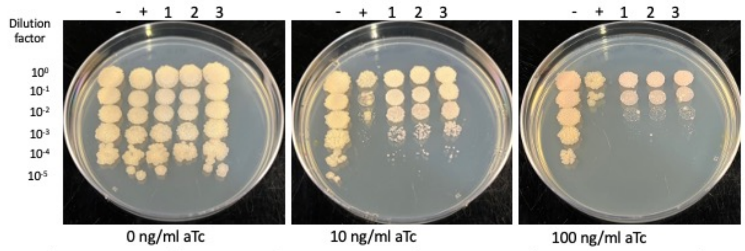

Lane	Plasmid name	Gene name, replicate	Toxic/Non-toxic	Colony color on 100 ng/ml aTc plate*
- Non-toxic control	pExTra03	Fruitloop *52* mutant	Non-toxic	+
+ Toxic control	pExTra02	Fruitloop *52*	Toxic	-
1	pExTra-Hammy84	Hammy *84* replicate 1	Toxic	+
2	pExTra-Hammy84	Hammy *84* replicate 2	Toxic	+
3	pExTra-Hammy84	Hammy *84* replicate 3	Toxic	+

*Key: NG (no growth) - (no pink color) +(faint pink color) ++(obvious pink color) +++ (dark pink color)

Gene *85*; Score *0*

Images taken after 3 days at 37 ºC

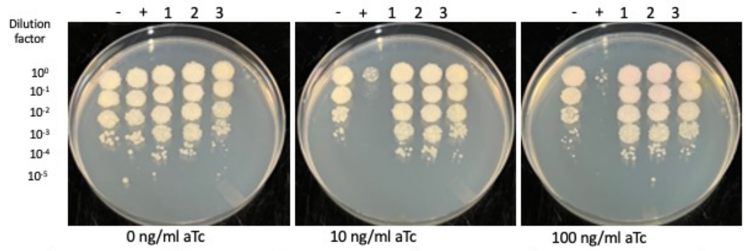

Lane	Plasmid name	Gene name, replicate	Toxic/Non-toxic	Colony color on 100 ng/ml aTc plate*
- Non-toxic control	pExTra03	Fruitloop *52* mutant	Non-toxic	+
+ Toxic control	pExTra02	Fruitloop *52*	Toxic	-
1	pExTra-Hammy85	Hammy *85* replicate 1	Non-toxic	+
2	pExTra-Hammy85	Hammy *85* replicate 2	Non-toxic	+
3	pExTra-Hammy85	Hammy *85* replicate 3	Non-toxic	+

*Key: NG (no growth) - (no pink color) +(faint pink color) ++(obvious pink color) +++ (dark pink color)

Gene *86*; Score *0*

Images taken after 3 days at 37 ºC

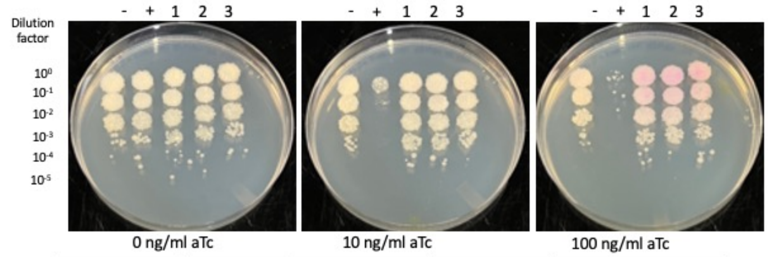

Lane	Plasmid name	Gene name, replicate	Toxic/Non-toxic	Colony color on 100 ng/ml aTc plate*
- Non-toxic control	pExTra03	Fruitloop *52* mutant	Non-toxic	-
+ Toxic control	pExTra02	Fruitloop *52*	Toxic	-
1	pExTra-Hammy86	Hammy *86* replicate 1	Non-toxic	++
2	pExTra-Hammy86	Hammy *86* replicate 2	Non-toxic	++
3	pExTra-Hammy86	Hammy *86* replicate 3	Non-toxic	++

*Key: NG (no growth) - (no pink color) +(faint pink color) ++(obvious pink color) +++ (dark pink color)

Gene *87*; Score *0*

Images taken after 3 days at 37 ºC

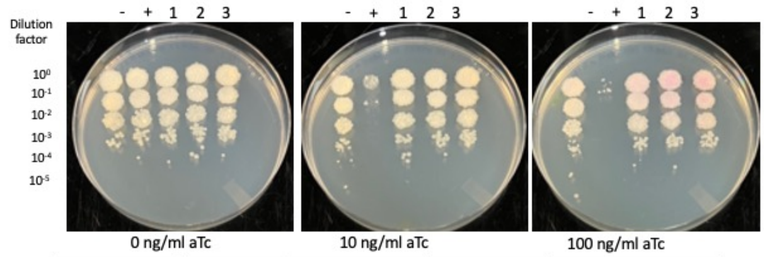

Lane	Plasmid name	Gene name, replicate	Toxic/Non-toxic	Colony color on 100 ng/ml aTc plate*
- Non-toxic control	pExTra03	Fruitloop *52* mutant	Non-toxic	-
+ Toxic control	pExTra02	Fruitloop *52*	Toxic	-
1	pExTra-Hammy87	Hammy *87* replicate 1	Non-toxic	+
2	pExTra-Hammy87	Hammy *87* replicate 2	Non-toxic	+
3	pExTra-Hammy87	Hammy *87* replicate 3	Non-toxic	++

*Key: NG (no growth) - (no pink color) +(faint pink color) ++(obvious pink color) +++ (dark pink color)

Gene *88*; Score *0*

Images taken after 3 days at 37 ºC

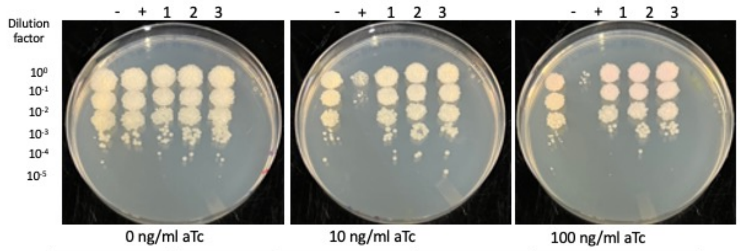

Lane	Plasmid name	Gene name, replicate	Toxic/Non-toxic	Colony color on 100 ng/ml aTc plate*
- Non-toxic control	pExTra03	Fruitloop *52* mutant	Non-toxic	+
+ Toxic control	pExTra02	Fruitloop *52*	Toxic	-
1	pExTra-Hammy88	Hammy *88* replicate 1	Non-toxic	+
2	pExTra-Hammy88	Hammy *88* replicate 2	Non-toxic	+
3	pExTra-Hammy88	Hammy *88* replicate 3	Non-toxic	+

*Key: NG (no growth) - (no pink color) +(faint pink color) ++(obvious pink color) +++ (dark pink color)

Gene *89*; Score *0*

Images taken after 3 days at 37 ºC

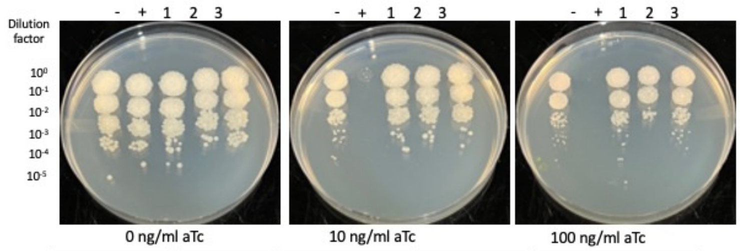

Lane	Plasmid name	Gene name, replicate	Toxic/Non-toxic	Colony color on 100 ng/ml aTc plate*
- Non-toxic control	pExTra03	Fruitloop *52* mutant	Non-toxic	-
+ Toxic control	pExTra02	Fruitloop *52*	Toxic	-
1	pExTra-Hammy89	Hammy *89* replicate 1	Non-toxic	-
2	pExTra-Hammy89	Hammy *89* replicate 2	Non-toxic	-
3	pExTra-Hammy89	Hammy *89* replicate 3	Non-toxic	-

*Key: NG (no growth) - (no pink color) +(faint pink color) ++(obvious pink color) +++ (dark pink color)

Gene *90*; Score *2*

Images taken after 4 days at 37 ºC

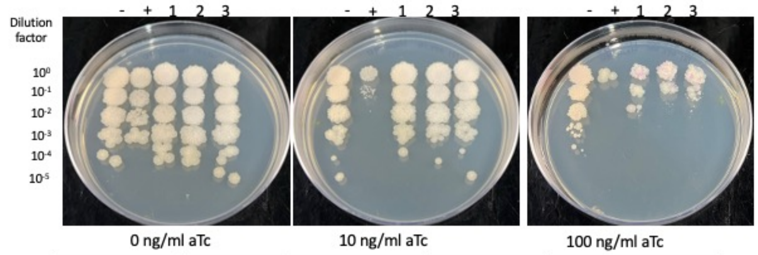

Lane	Plasmid name	Gene name, replicate	Toxic/Non-toxic	Colony color on 100 ng/ml aTc plate*
- Non-toxic control	pExTra03	Fruitloop *52* mutant	Non-toxic	+
+ Toxic control	pExTra02	Fruitloop *52*	Toxic	-
1	pExTra-Hammy90	Hammy *90* replicate 1	Toxic	-
2	pExTra-Hammy90	Hammy *90* replicate 2	Toxic	-
3	pExTra-Hammy90	Hammy *90* replicate 3	Toxic	-

*Key: NG (no growth) - (no pink color) +(faint pink color) ++(obvious pink color) +++ (dark pink color)

Images taken after 3 days at 37 ºC

Gene *91*; Score *0*

- + 1 2 3   - + 1 2 3   - + 1 2 3

Dilution factor: 10^0, 10^{-1}, 10^{-2}, 10^{-3}, 10^{-4}, 10^{-5}

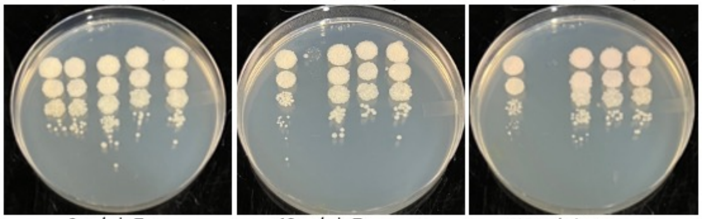

0 ng/ml aTc   10 ng/ml aTc   100 ng/ml aTc

Lane	Plasmid name	Gene name, replicate	Toxic/Non-toxic	Colony color on 100 ng/ml aTc plate*
- Non-toxic control	pExTra03	Fruitloop *52* mutant	Non-toxic	+
+ Toxic control	pExTra02	Fruitloop *52*	Toxic	NG
1	pExTra-Hammy91	Hammy *91* replicate 1	Non-toxic	+
2	pExTra-Hammy91	Hammy *91* replicate 2	Non-toxic	+
3	pExTra-Hammy91	Hammy *91* replicate 3	Non-toxic	+

*Key: NG (no growth)   - (no pink color)   +(faint pink color)   ++(obvious pink color)   +++ (dark pink color)

Images taken after 4 days at 37 ºC

Gene *92*; Score *2*

- + 1 2 3   - + 1 2 3   - + 1 2 3

Dilution factor: 10^0, 10^{-1}, 10^{-2}, 10^{-3}, 10^{-4}, 10^{-5}

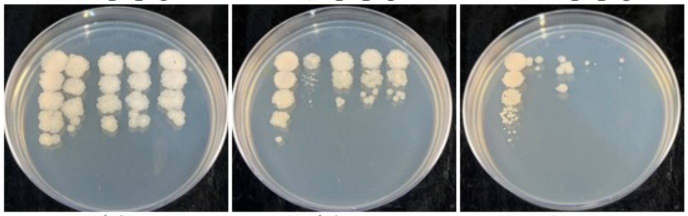

0 ng/ml aTc   10 ng/ml aTc   100 ng/ml aTc

Lane	Plasmid name	Gene name, replicate	Toxic/Non-toxic	Colony color on 100 ng/ml aTc plate*
- Non-toxic control	pExTra03	Fruitloop *52* mutant	Non-toxic	+
+ Toxic control	pExTra02	Fruitloop *52*	Toxic	-
1	pExTra-Hammy92	Hammy *92* replicate 1	Toxic	-/NG
2	pExTra-Hammy92	Hammy *92* replicate 2	Toxic	-/NG
3	pExTra-Hammy92	Hammy *92* replicate 3	Toxic	NG

*Key: NG (no growth)   - (no pink color)   +(faint pink color)   ++(obvious pink color)   +++ (dark pink color)

Images taken after 4 days at 37 ºC

Gene *93*; Score *0*

- + 1 2 3   - + 1 2 3   - + 1 2 3

Dilution factor: 10^0, 10^{-1}, 10^{-2}, 10^{-3}, 10^{-4}, 10^{-5}

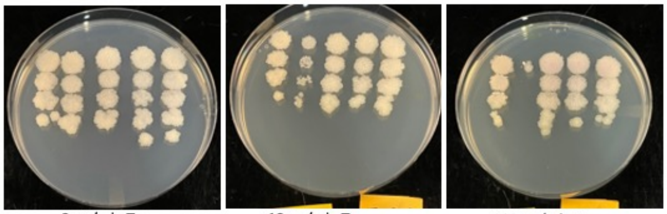

0 ng/ml aTc   10 ng/ml aTc   100 ng/ml aTc

Lane	Plasmid name	Gene name, replicate	Toxic/Non-toxic	Colony color on 100 ng/ml aTc plate*
- Non-toxic control	pExTra03	Fruitloop *52* mutant	Non-toxic	+
+ Toxic control	pExTra02	Fruitloop *52*	Toxic	NG
1	pExTra-Hammy93	Hammy *93* replicate 1	Non-toxic	+
2	pExTra-Hammy93	Hammy *93* replicate 2	Non-toxic	+
3	pExTra-Hammy93	Hammy *93* replicate 3	Non-toxic	+

*Key: NG (no growth)   - (no pink color)   +(faint pink color)   ++(obvious pink color)   +++ (dark pink color)

Images taken after 4 days at 37 ºC

Gene *94*; Score *0*

- + 1 2 3   - + 1 2 3   - + 1 2 3

Dilution factor: 10^0, 10^{-1}, 10^{-2}, 10^{-3}, 10^{-4}, 10^{-5}

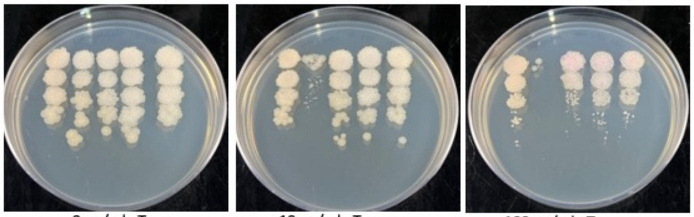

0 ng/ml aTc   10 ng/ml aTc   100 ng/ml aTc

Lane	Plasmid name	Gene name, replicate	Toxic/Non-toxic	Colony color on 100 ng/ml aTc plate*
- Non-toxic control	pExTra03	Fruitloop *52* mutant	Non-toxic	+
+ Toxic control	pExTra02	Fruitloop *52*	Toxic	-
1	pExTra-Hammy94	Hammy *94* replicate 1	Non-toxic	+
2	pExTra-Hammy94	Hammy *94* replicate 2	Non-toxic	+
3	pExTra-Hammy94	Hammy *94* replicate 3	Non-toxic	+

*Key: NG (no growth)   - (no pink color)   +(faint pink color)   ++(obvious pink color)   +++ (dark pink color)

Images taken after 4 days at 37 ºC

Gene *95*; Score *0*

- + 1 2 3   - + 1 2 3   - + 1 2 3

Dilution factor: 10^0, 10^{-1}, 10^{-2}, 10^{-3}, 10^{-4}, 10^{-5}

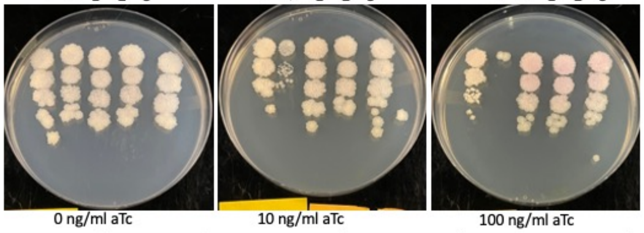

0 ng/ml aTc   10 ng/ml aTc   100 ng/ml aTc

Lane	Plasmid name	Gene name, replicate	Toxic/Non-toxic	Colony color on 100 ng/ml aTc plate*
- Non-toxic control	pExTra03	Fruitloop *52* mutant	Non-toxic	+
+ Toxic control	pExTra02	Fruitloop *52*	Toxic	NG
1	pExTra-Hammy95	Hammy *95* replicate 1	Non-toxic	++
2	pExTra-Hammy95	Hammy *95* replicate 2	Non-toxic	++
3	pExTra-Hammy95	Hammy *95* replicate 3	Non-toxic	++

*Key: NG (no growth)   - (no pink color)   +(faint pink color)   ++(obvious pink color)   +++ (dark pink color)

Supplemental Table 1: DNA oligos used in this study

Oligo Name	Oligo Sequence (5' to 3')
**oHammy1F**	ATGCGGAGGAATCACTTCCATATGATCGACACCTACCGCACG
**oHammy1R**	TGCAGGATCCGACTCGAGTGTCGACTCACTCTGCCTCGATGAAGTC
**oHammy2F**	ATGCGGAGGAATCACTTCCATATGACGCCGACCGTCGGGCG
**oHammy2R**	TGCAGGATCCGACTCGAGTGTCGACTCATGCGCGCGGGGGCCAGT
**oHammy3F**	ATGCGGAGGAATCACTTCCATATGAGGCCGCGGCCCGTCGG
**oHammy3R**	TGCAGGATCCGACTCGAGTGTCGACTCATCGGGGTGAGTCGGACAG
**oHammy5F**	ATGCGGAGGAATCACTTCCATATGCGTGCACCGAGCACCGTC
**oHammy5R**	TGCAGGATCCGACTCGAGTGTCGACTCAGCCATCGCTGCCACCGCC
**oHammy6F**	ATGCGGAGGAATCACTTCCATATGGCTGACGACAAGCTCGAC
**oHammy6R**	TGCAGGATCCGACTCGAGTGTCGACTCAGACGTCCGAACCATCGAA
**oHammy7F**	ATGCGGAGGAATCACTTCCATATGGTTCGGACGTCTGAGCCG
**oHammy7R**	TGCAGGATCCGACTCGAGTGTCGACTCACACGAACATGGCGCCACC
**oHammy8F**	ATGCGGAGGAATCACTTCCATATGATTCCCGCTGCCTATGAC
**oHammy8R**	TGCAGGATCCGACTCGAGTGTCGACTCACTGAGTCGGGCGAGCGCC
**oHammy9F**	ATGCGGAGGAATCACTTCCATATGACCGAGCCGACGGCGGTA
**oHammy9R**	TGCAGGATCCGACTCGAGTGTCGACTCATTGGTTGTAGTCCGGTGTC
**oHammy10F**	ATGCGGAGGAATCACTTCCATATGACGCCCGAGCAGCAGTCTT
**oHammy10R**	TGCAGGATCCGACTCGAGTGTCGACTCAGTCGGCGTTAGCCCGCAGG
**oHammy11F**	ATGCGGAGGAATCACTTCCATATGGGAGACACCGACACCGGC
**oHammy11R**	TGCAGGATCCGACTCGAGTGTCGACTCACTCCCCAGAGCGCAAGCG
**oHammy12F**	ATGCGGAGGAATCACTTCCATATGGCTGACATTTCACGCGCC
**oHammy12R**	TGCAGGATCCGACTCGAGTGTCGACTCAGCTACCGCCCTGCGCTGC
**oHammy13F**	ATGCGGAGGAATCACTTCCATATGGCACTGGCGACGACAGCG
**oHammy13R**	TGCAGGATCCGACTCGAGTGTCGACTCAGTAACGCTCACTGCCCA
**oHammy14F**	ATGCGGAGGAATCACTTCCATATGTTCCCGACGCCTCACACG
**oHammy14R**	TGCAGGATCCGACTCGAGTGTCGACCTATACGCCATCGTCGCCCCCCT
**oHammy15F**	ATGCGGAGGAATCACTTCCATATGGCGTATAGGCCCCTCGAT
**oHammy15R**	TGCAGGATCCGACTCGAGTGTCGACTCACAGCAGCCCACCCCTCGG
**oHammy16F**	ATGCGGAGGAATCACTTCCATATGACGGTCCTACTGCCTCCC
**oHammy16R**	TGCAGGATCCGACTCGAGTGTCGACTCAGCCAGGCCGTAGGCCGAT
**oHammy17F**	ATGCGGAGGAATCACTTCCATATGACCGGACCCGTTACACCG
**oHammy17R**	TGCAGGATCCGACTCGAGTGTCGACTCAAGCGGCGACGACCTGACCG
**oHammy18F**	ATGCGGAGGAATCACTTCCATATGGCAACCGCCAAGAGCAAG
**oHammy18R**	TGCAGGATCCGACTCGAGTGTCGACCTACCTGCCGCGCCGCCGCGC
**oHammy20F**	ATGCGGAGGAATCACTTCCATATGAGCGCAACGTATTACCTCAC
**oHammy20R**	TGCAGGATCCGACTCGAGTGTCGACTCAGGTGTAGCGCCTGCGGCTGT
**oHammy21F**	ATGCGGAGGAATCACTTCCATATGACCGGCACTGGTATTCACG

**oHammy21R**	TGCAGGATCCGACTCGAGTGTCGACTCAGGACCACGCCATGCGGTAG
**oHammy22F**	ATGCGGAGGAATCACTTCCATATGTACGTCAAGAACGGCCGCA
**oHammy22R**	TGCAGGATCCGACTCGAGTGTCGACCTAGAACATGTCTCCTGATCCAAAC
**oHammy23F**	ATGCGGAGGAATCACTTCCATATGGATCTGCCAGCCCTGCCA
**oHammy23R**	TGCAGGATCCGACTCGAGTGTCGACTCAGCTGTTGGCGGGCTCGTC
**oHammy24F**	ATGCGGAGGAATCACTTCCATATGGCCGAGGTTGAGCAGCGG
**oHammy24R**	TGCAGGATCCGACTCGAGTGTCGACTCAGCTCCCTTGTGGGACAACGA
**oHammy25F**	ATGCGGAGGAATCACTTCCATATGCCGTACACCAAGAGCTACC
**oHammy25R**	TGCAGGATCCGACTCGAGTGTCGACTCAGTCACCCCCCGCCTTCACC
**oHammy26F**	ATGCGGAGGAATCACTTCCATATGCCGTCCATGTATGACCGGC
**oHammy26R**	TGCAGGATCCGACTCGAGTGTCGACTCACGCCAGACTCCTATTCAGT
**oHammy27F**	ATGCGGAGGAATCACTTCCATATGACCGGATGGACACCAGACC
**oHammy27R**	TGCAGGATCCGACTCGAGTGTCGACTCACTGGTAGGCGCGGAACCATG
**oHammy28F**	ATGCGGAGGAATCACTTCCATATGGCTGCCACGAATCAGTTCAA
**oHammy28R**	TGCAGGATCCGACTCGAGTGTCGACTCAGACGGGATCGCCGTACGTGT
**oHammy29F**	ATGCGGAGGAATCACTTCCATATGGCTGAAAAGGTACTGCCTTAC
**oHammy29R**	TGCAGGATCCGACTCGAGTGTCGACTCACAATGCCCCCTTCTTGAGC
**oHammy30F**	ATGCGGAGGAATCACTTCCATATGAGCAAGCCCATGCTGCTGA
**oHammy30R**	TGCAGGATCCGACTCGAGTGTCGACTCACGCCGCCCGTGCGCGCAT
**oHammy31F**	ATGCGGAGGAATCACTTCCATATGAGCAAAACCGTTGAGAACAT
**oHammy31R**	TGCAGGATCCGACTCGAGTGTCGACTCACGTCGTCGCCGCCGTGTG
**oHammy32F**	ATGCGGAGGAATCACTTCCATATGAGCGGCGAGTCGGCACTA
**oHammy32R**	TGCAGGATCCGACTCGAGTGTCGACTCACGCCTGCACCATCAGCGA
**oHammy33F**	ATGCGGAGGAATCACTTCCATATGAGCCTGGCTGACCGTCTC
**oHammy33R**	TGCAGGATCCGACTCGAGTGTCGACTCAGACAGAGACACGGGCGCC
**oHammy34F**	ATGCGGAGGAATCACTTCCATATGTCTCTGTCTGATCGCCTC
**oHammy34R**	TGCAGGATCCGACTCGAGTGTCGACTCACAGCACGCCCAATCGGGA
**oHammy35F**	ATGCGGAGGAATCACTTCCATATGATGCGGTGCGCTGATCGG
**oHammy35R**	TGCAGGATCCGACTCGAGTGTCGACTCAGCTGCCGCGGGTCGCCTG
**oHammy36F**	ATGCGGAGGAATCACTTCCATATGAGCACGCCCACAATGACA
**oHammy36R**	TGCAGGATCCGACTCGAGTGTCGACTCACTTCGCCCGGTCCTGCTT
**oHammy37F**	ATGCGGAGGAATCACTTCCATATGAGCGACGCCGCCGCGGTC
**oHammy37R**	TGCAGGATCCGACTCGAGTGTCGACTCATGGCGCAACCACGTCCCG
**oHammy38F**	ATGCGGAGGAATCACTTCCATATGACGCTGCAGCGTAAGCCC
**oHammy38R**	TGCAGGATCCGACTCGAGTGTCGACTCACTTCGGCGAACGCTTCGCGT
**oHammy39F**	ATGCGGAGGAATCACTTCCATATGCAAGAGCACTTTTACCTCGG
**oHammy39R**	TGCAGGATCCGACTCGAGTGTCGACTCAGGCGACGTCGAGGTCGAACA
**oHammy40F**	ATGCGGAGGAATCACTTCCATATGCGCCTGCGGCCGGGCCGC
**oHammy40R**	TGCAGGATCCGACTCGAGTGTCGACTCAGCGGCCGTCGAGCGCCCG

**oHammy41F**	ATGCGGAGGAATCACTTCCATATGCACCCAAAGGTGTACCCA
**oHammy41R**	TGCAGGATCCGACTCGAGTGTCGACTCAGGCCCCGAGGTCGTCGGC
**oHammy42F**	ATGCGGAGGAATCACTTCCATATGGTGGCAAAAGCTCGGCGC
**oHammy42R**	TGCAGGATCCGACTCGAGTGTCGACTCACGCCGCCAATAGGTCGCC
**oHammy43F**	ATGCGGAGGAATCACTTCCATATGCGCAAATTGTCGGGCTCG
**oHammy43R**	TGCAGGATCCGACTCGAGTGTCGACTCAGTCGGGCTCAGCCTCGGGTG
**oHammy44F**	ATGCGGAGGAATCACTTCCATATGACCGTCCTACTGTTTGCC
**oHammy44R**	TGCAGGATCCGACTCGAGTGTCGACTCAGCTGACCGGCTCGGGCGGCTG
**oHammy45F**	ATGCGGAGGAATCACTTCCATATGGACGACAGCAACAAGTCGCT
**oHammy45R**	TGCAGGATCCGACTCGAGTGTCGACTCACAGCCCCGAGACGCCGTTGC
**oHammy46F**	ATGCGGAGGAATCACTTCCATATGGGCGACAACGGAATCCGCG
**oHammy46R**	TGCAGGATCCGACTCGAGTGTCGACTCACGCGGCGGCCTCGGCAAG
**oHammy47F**	ATGCGGAGGAATCACTTCCATATGATCCCGCCGGTTGTCGTC
**oHammy47R**	TGCAGGATCCGACTCGAGTGTCGACTCAGGCCCCTAACTGGTCGAC
**oHammy48F**	ATGCGGAGGAATCACTTCCATATGTCACTTGCACGCCATATGG
**oHammy48R**	TGCAGGATCCGACTCGAGTGTCGACTCAACCCGCGCGATCCATGTCG
**oHammy49F**	ATGCGGAGGAATCACTTCCATATGCCGTGCAAAATTCTACGTC
**oHammy49R**	TGCAGGATCCGACTCGAGTGTCGACTCACGACACCGCCCGCGCGAG
**oHammy50F**	ATGCGGAGGAATCACTTCCATATGAGCCCCGCACCGTACTGC
**oHammy50R**	TGCAGGATCCGACTCGAGTGTCGACTCACAGCGACCACACCCCGAC
**oHammy51F**	ATGCGGAGGAATCACTTCCATATGAGCTTCTGCATTGCCTACGG
**oHammy51R**	TGCAGGATCCGACTCGAGTGTCGACTCACGCCTGCACCGCCTGCCGTG
**oHammy52F**	ATGCGGAGGAATCACTTCCATATGAGGGCTGCACAGATCGACGA
**oHammy52R**	TGCAGGATCCGACTCGAGTGTCGACTCACTTGTCGGCCTCGCTTTCGG
**oHammy53F**	ATGCGGAGGAATCACTTCCATATGAACGCCCTTACCCTGCCAGA
**oHammy53R**	TGCAGGATCCGACTCGAGTGTCGACTCATGCGGCGGCCCCCTCACG
**oHammy54F**	ATGCGGAGGAATCACTTCCATATGAGCGATCAGCCAATGTGC
**oHammy54R**	TGCAGGATCCGACTCGAGTGTCGACTCATCCCTTTGTTTCGTTCGCAG
**oHammy55F**	ATGCGGAGGAATCACTTCCATATGCACGCATTCATCAAGGCC
**oHammy55R**	TGCAGGATCCGACTCGAGTGTCGACTCACGGGCGATCGACGTCGGG
**oHammy56F**	ATGCGGAGGAATCACTTCCATATGACCGACAGCAAGCGCCCG
**oHammy56R**	TGCAGGATCCGACTCGAGTGTCGACTCAGGCATTGCTTGCCCTTTCG
**oHammy57F**	ATGCGGAGGAATCACTTCCATATGCCTGACATTTCAGAGGTC
**oHammy57R**	TGCAGGATCCGACTCGAGTGTCGACTCATCCCTTGTCCGTGTCGACCAG
**oHammy58F**	ATGCGGAGGAATCACTTCCATATGGCAAGGCAACTGATTGTCGTC
**oHammy58R**	TGCAGGATCCGACTCGAGTGTCGACTCACAGGGCCGCCGCCTTCGT
**oHammy59F**	ATGCGGAGGAATCACTTCCATATGAGCAATTTCGCGCACGTG
**oHammy59R**	TGCAGGATCCGACTCGAGTGTCGACTCATCGCTGCGCACCGCCCAT
**oHammy60F**	ATGCGGAGGAATCACTTCCATATGAGCGGCAACGCAGGCTTT

**oHammy60R**	TGCAGGATCCGACTCGAGTGTCGACTCACTTGGCGCCGCCCTCGCA
**oHammy61F**	ATGCGGAGGAATCACTTCCATATGAGCCACTACATGCGCACG
**oHammy61R**	TGCAGGATCCGACTCGAGTGTCGACTCAGACGACTACAACGCCGAA
**oHammy62F**	ATGCGGAGGAATCACTTCCATATGCAAGATGACAGCCCCCGA
**oHammy62R**	TGCAGGATCCGACTCGAGTGTCGACTCACGGCTGCGCCTCGTCGAG
**oHammy63F**	ATGCGGAGGAATCACTTCCATATGAGCGCCCGGTCGACGTTC
**oHammy63R**	TGCAGGATCCGACTCGAGTGTCGACTCACGGGCATTCACCTTTCGG
**oHammy64F**	ATGCGGAGGAATCACTTCCATATGAGCCGTCGGCACAACTGC
**oHammy64R**	TGCAGGATCCGACTCGAGTGTCGACTCAGGTCCCGTCGTAGTAGTCGG
**oHammy65F**	ATGCGGAGGAATCACTTCCATATGAGCAACGATTCGTACGGATT
**oHammy65R**	TGCAGGATCCGACTCGAGTGTCGACTCACTTGACCATGCCGAGCTTCT
**oHammy66F**	ATGCGGAGGAATCACTTCCATATGCTGACCGTCTACACGACCGG
**oHammy66R**	TGCAGGATCCGACTCGAGTGTCGACTCAGCGGGCCTCGATCGCGGC
**oHammy67F**	ATGCGGAGGAATCACTTCCATATGCCCGTCGATACCCGGCTG
**oHammy67R**	TGCAGGATCCGACTCGAGTGTCGACTCACAGTGCAAACAGCCCATC
**oHammy68F**	ATGCGGAGGAATCACTTCCATATGAACGGCCTAACTGACCTGC
**oHammy68R**	TGCAGGATCCGACTCGAGTGTCGACTCACCACCGCGGGGCCTCGTTC
**oHammy69F**	ATGCGGAGGAATCACTTCCATATGACTGACCACACTCTCGACC
**oHammy69R**	TGCAGGATCCGACTCGAGTGTCGACTCAGGCATTAGCCGCCCGCCAT
**oHammy70F**	ATGCGGAGGAATCACTTCCATATGCGCACTCGTTTTGAGGCCC
**oHammy70R**	TGCAGGATCCGACTCGAGTGTCGACTCACGACGGCCGCACCTCCGTG
**oHammy71F**	ATGCGGAGGAATCACTTCCATATGACGAGTTTGCCGGAAATCG
**oHammy71R**	TGCAGGATCCGACTCGAGTGTCGACTCATCCCGTGTCCGAAACTGACGG
**oHammy72F**	ATGCGGAGGAATCACTTCCATATGCCTTTGAAGCGCAACAGATTA
**oHammy72R**	TGCAGGATCCGACTCGAGTGTCGACTCACTTCGCGCCCTCGTTCAGGTG
**oHammy73F**	ATGCGGAGGAATCACTTCCATATGAAGCGCGTCAAGACTGTTC
**oHammy73R**	TGCAGGATCCGACTCGAGTGTCGACTCACGCTGCGGCGTTCGCGCGG
**oHammy74F**	ATGCGGAGGAATCACTTCCATATGAACCGCAATCTCATTCTCG
**oHammy74R**	TGCAGGATCCGACTCGAGTGTCGACTCAGTCGCTGGCACCTCCCCCG
**oHammy75F**	ATGCGGAGGAATCACTTCCATATGAGCGAAGTCATCGACTACAG
**oHammy75R**	TGCAGGATCCGACTCGAGTGTCGACTCAGCGAGTTGCGTACTTGCG
**oHammy76F**	ATGCGGAGGAATCACTTCCATATGCACAACACCCACGTTTAC
**oHammy76R**	TGCAGGATCCGACTCGAGTGTCGACTCAGTCGGCCGCCATGTGCGCCA
**oHammy77F**	ATGCGGAGGAATCACTTCCATATGACCGAAAACATCACCCGC
**oHammy77R**	TGCAGGATCCGACTCGAGTGTCGACTCAGGACTCGATGCGGTCGTG
**oHammy78F**	ATGCGGAGGAATCACTTCCATATGAACACGAAAGATCCGAGG
**oHammy78R**	TGCAGGATCCGACTCGAGTGTCGACTCACACCGCCACCGCCTCGAC

**oHammy79F**	ATGCGGAGGAATCACTTCCATATGAGCGCACTCTCTGTCGCT
**oHammy79R**	TGCAGGATCCGACTCGAGTGTCGACTCACCAGAGCGACCCCAGCCC
**oHammy80F**	ATGCGGAGGAATCACTTCCATATGATCGCGCTCACTGAAATGC
**oHammy80R**	TGCAGGATCCGACTCGAGTGTCGACTCACATCACGCACACATAGCCG
**oHammy81F**	ATGCGGAGGAATCACTTCCATATGATGCTGTCCGTTGAGCCGG
**oHammy81R**	TGCAGGATCCGACTCGAGTGTCGACTCATTTCGGGTCGGGTCGGTCA
**oHammy82F**	ATGCGGAGGAATCACTTCCATATGCGAGGACTGATCGACAGG
**oHammy82R**	TGCAGGATCCGACTCGAGTGTCGACTCAGCCGATACAGTCGCCGCA
**oHammy83F**	ATGCGGAGGAATCACTTCCATATGTTGGCACCTCAACAGATCAG
**oHammy83R**	TGCAGGATCCGACTCGAGTGTCGACTCACTGCCCCTTCACGTTCAAG
**oHammy84F**	ATGCGGAGGAATCACTTCCATATGAGCACAGAAGGCTTTTCGC
**oHammy84R**	TGCAGGATCCGACTCGAGTGTCGACTCACCCATGCCGCAACGCCTGC
**oHammy85F**	ATGCGGAGGAATCACTTCCATATGGGTGAGTCGGCGCTGTTC
**oHammy85R**	TGCAGGATCCGACTCGAGTGTCGACTCACGCTGCCACCGCCGCAGG
**oHammy86F**	ATGCGGAGGAATCACTTCCATATGAGCGGCGATCTGCGCGAG
**oHammy86R**	TGCAGGATCCGACTCGAGTGTCGACTCATCGCCTCGCCCCCGTCCC
**oHammy87F**	ATGCGGAGGAATCACTTCCAATGAGCGTCGCGGTTGTGGCG
**oHammy87R**	TGCAGGATCCGACTCGAGTGTCGACTCAGAACGGTGGCGGCAAGCT
**oHammy88F**	ATGCGGAGGAATCACTTCCATATGCAGGAATACACCAAGGCC
**oHammy88R**	TGCAGGATCCGACTCGAGTGTCGACTCACTTGCCCTTGGGCGCCTTG
**oHammy89F**	ATGCGGAGGAATCACTTCCATATGCAGAACACAATCGCCGCC
**oHammy89R**	TGCAGGATCCGACTCGAGTGTCGACTCAGGCGATTTCTGACCACTC
**oHammy90F**	ATGCGGAGGAATCACTTCCATATGACCGACACGATTCACGCA
**oHammy90R**	TGCAGGATCCGACTCGAGTGTCGACTCAGGCCGCCGCGGCGCGCCG
**oHammy91F**	ATGCGGAGGAATCACTTCCATATGTACGCATGGATCAATGGTCAG
**oHammy91R**	TGCAGGATCCGACTCGAGTGTCGACTCAGCAGCCGTGCAGCATCTTG
**oHammy92F**	ATGCGGAGGAATCACTTCCATATGAGCACGACCGTTACCTACC
**oHammy92R**	TGCAGGATCCGACTCGAGTGTCGACTCAGGCCGCCGCGGGCTCGAC
**oHammy93F**	ATGCGGAGGAATCACTTCCATATGGCTCTAATCAGCCCTGAG
**oHammy93R**	TGCAGGATCCGACTCGAGTGTCGACTCAGAGCTTGAAGGGGAAGGGC
**oHammy94F**	ATGCGGAGGAATCACTTCCATATGGCACAAGACAGCACCGAG
**oHammy94R**	TGCAGGATCCGACTCGAGTGTCGACTCAGTCGAGCGGTTCGTACTT
**oHammy95F**	ATGCGGAGGAATCACTTCCATATGAACGAAGCTCGCAACACTG
**oHammy95R**	TGCAGGATCCGACTCGAGTGTCGACTCAGGGCCGCCAGACCCGAGCG
**oHammy9_internal1**	GGTCACGATCACTGCAAGTGC
**oHammy9_internal2**	GAAACCGCTACGCGCG
**oHammy20_internal1**	CTGCCTCTGCGGTGGC

**oHammy20_internal2**	CGACTGGTCGACAAGGTGC
**oHammy20_internal3**	CACCTTGTCGTGAGCCCG
**oHammy20_internal4**	GTCGTTCGGCCGGTAACC
**oHammy22_internal1**	CGATCCTGGCCTTGCCAAC
**oHammy26_internal1**	CAGCCAGCGGCTCGATC
**oHammy26_internal2**	GACACGTTGCCGGTATCCG
**oHammy26_internal3**	CGATCGGGCAGCAGCAAG
**oHammy29_internal1**	CGTTCGACCGTTACGCC
**oHammy68_internal1**	CGTCACGATCGACGG
**oHammy68_internal2**	GGTTGCCCATCAAGAACAGC
**oHammy68_internal3**	GGTCACGTCGCAACCAAG
**pExTra_seqF**	GTACCCGTGTGTACGACCAGC
**pExTra_universalR**	CCCTTCGAGACCATAGATCTGTTCC
